# Robot-Assisted Transcranial Doppler Versus Transthoracic Echocardiography for Right to Left Shunt Detection

**DOI:** 10.1161/STROKEAHA.123.043380

**Published:** 2023-10-05

**Authors:** Mark N. Rubin, Ruchir Shah, Thomas Devlin, Teddy S. Youn, Michael F. Waters, John J. Volpi, Aaron Stayman, Colleen M. Douville, Ted Lowenkopf, Georgios Tsivgoulis, Andrei V. Alexandrov

**Affiliations:** Edward Hines, Jr. Veterans Affairs Medical Center, IL (M.N.R.).; CHI Memorial Hospital, Chattanooga, TN (R.S., T.D.).; Barrow Neurological Institute, Phoenix, AZ (T.S.Y., M.F.W.).; Houston Methodist Research Institute, TX (J.J.V.).; Swedish Medical Center, Seattle, WA (A.S., C.M.D.).; Providence Brain and Spine Institute, Portland, OR (T.L.).; Second Department of Neurology, National and Kapodistrian University of Athens, “Attikon” University Hospital, Greece (G.T.).; Department of Neurology, Banner University Hospital, University of Arizona College of Medicine, Phoenix (A.V.A.).

**Keywords:** echocardiography, embolic stroke, embolism, ischemic stroke, patent foramen ovale, robotics, ultrasound

## Abstract

**BACKGROUND:**

Right to left shunt (RLS), including patent foramen ovale, is a recognized risk factor for stroke. RLS/patent foramen ovale diagnosis is made by transthoracic echocardiography (TTE), which is insensitive, transesophageal echocardiography, which is invasive, and transcranial Doppler (TCD), which is noninvasive and accurate but scarce.

**METHODS:**

We conducted a prospective, single-arm device clinical trial of robot-assisted TCD (raTCD) versus TTE for RLS diagnosis at 6 clinical sites in patients who presented with an event suspicious for embolic cerebrovascular ischemia from October 6, 2020 to October 20, 2021. raTCD was performed with standard TCD bubble study technique. TTE bubble study was performed per local standards. The primary outcome was rate of RLS detection by raTCD versus TTE.

**RESULTS:**

A total of 154 patients were enrolled, 129 evaluable (intent to scan) and 121 subjects had complete data per protocol. In the intent to scan cohort, mean age was 60±15 years, 47% were women, and all qualifying events were diagnosed as ischemic stroke or transient ischemic attack. raTCD was positive for RLS in 82 subjects (64%) and TTE was positive in 26 (20%; absolute difference 43.4% [95% CI, 35.2%–52.0%]; *P*<0.001). On prespecified secondary analysis, large RLS was detected by raTCD in 35 subjects (27%) versus 13 (10%) by TTE (absolute difference 17.0% [95% CI, 11.5%–24.5%]; *P*<0.001). There were no serious adverse events.

**CONCLUSIONS:**

raTCD was safe and ≈3 times more likely to diagnose RLS than TTE. TTE completely missed or underdiagnosed two thirds of large shunts diagnosed by raTCD. The raTCD device, used by health professionals with no prior TCD training, may allow providers to achieve the known sensitivity of TCD for RLS and patent foramen ovale detection without the need for an experienced operator to perform the test. Pending confirmatory studies, TCD appears to be the superior screen for RLS compared with TTE (funded by NeuraSignal).

**REGISTRATION:**

URL: https://www.clinicaltrials.gov; Unique identifier: NCT04604015.

**See related article, p** 2851

Acute ischemic stroke patients should be considered for investigation of a source of cerebral embolism, and one element of the investigation is to screen for a right to left shunt (RLS), the most common source being a patent foramen ovale (PFO).^1^ PFO is estimated to be present in ≈25% of the general population^2^ but is overrepresented in the ischemic stroke population, especially those who are <60 years of age and without traditional vascular risk factors, where prevalence is estimated to be as high as 30% to 50%.^3,4^ A RLS can be a conduit for a peripheral venous thrombus to embolize the cerebral arterial circulation (paradoxical embolization)^3^ or, as is the case with PFO, particularly with an atrial septal aneurysm,^5^ may itself be the thrombogenic source of acute cerebral ischemia.^6^ The presence or absence of RLS impacts the choice of stroke risk reduction therapies and prognosis.^7–12^ Therefore, effective screening for RLS is a sine qua non of a thorough evaluation for embolic stroke with no other identified source to avoid exposing a patient to excess risk of stroke recurrence due to undertreatment.

Transthoracic echocardiography (TTE) with agitated saline contrast is the most common screening diagnostic for RLS but has been shown to have a low sensitivity of ≈45% for PFO^13,14^ as compared with transesophageal echocardiography (TEE), making it a poor screening examination despite its widespread availability and noninvasive nature. TEE is the nonsurgical gold standard for PFO diagnosis^14,15^ but is invasive, requires sedating medications that can limit Valsalva effort by the patient and negatively impact test sensitivity,^15^ and does not directly visualize extracardiac shunting. Transcranial Doppler (TCD) is very sensitive (96%) and specific (92%) for the diagnosis of PFO as compared with TEE,^13^ is noninvasive, can be performed at the point of care, allows for both calibrated Valsalva^16^ and body positioning^17^ to increase sensitivity, and has established shunt grading schema^16,18,19^ that can assist in RLS evaluation and management, but is operator-dependent and limited by the availability of sonographers and physicians with expertise.^20^

Recently, robot-assisted TCD (raTCD) technology,^21^ some with machine-learning-enhanced signal detection algorithms,^22^ has been introduced to clinical research and practice to help mitigate barriers to TCD performance. More specifically, raTCD can detect and maintain optimal cerebral blood flow velocity signals for embolic monitoring autonomously, with potential to expand the availability but maintain the high diagnostic accuracy of TCD for RLS diagnosis. However, the diagnostic accuracy of raTCD has never been prospectively tested against the most common RLS screening examination, TTE.

## METHODS

### Trial Oversight and Funding

This study comports with the Transparency and Openness Promotion Guidelines for authors publishing in the American Heart Association Journal, and the data sets can be made available by reasonable request to the corresponding author.

The study was a multicenter (conducted within 6 clinical sites), prospective, single-arm, nonsignificant risk, consecutively enrolled diagnostic yield device clinical trial. The trial was run between October 6, 2020 and October 21, 2021. Specific details about methodology, including prespecified outcomes, were published previously.^23^ This trial was registered with ClinicalTrials.gov (https://www.clinicaltrials.gov; Unique identifier: NCT04604015). The trial and protocol were designed by an academic steering committee. The funders, NeuraSignal, Inc, had no influence on the final design or conduct of the trial, in the writing of the article, or in the decision to submit it for publication. The trial protocol (available in full in the Supplemental Material), and informed consent forms were reviewed and approved by central and institutional internal review boards as appropriate at each study site. The trial was performed in accordance with the principles of the Declaration of Helsinki. The trial was designed to align with STARD (Standards for Reporting of Diagnostic Accuracy Studies),^24^ standards of device accuracy trials and the checklist available with Supplemental Material. The authors assume responsibility for the accuracy and completeness of the data and analyses, and for the fidelity of the trial and this report to the protocol.

### Patients

The trial included adult (≥18 years) patients who experienced a clinical episode that, in the opinion of the treatment team, included an embolic acute neurovascular episode (eg, ischemic stroke or transient ischemic attack) on the differential diagnosis prompting patient referral for a TTE with agitated saline bubble contrast as part of routine clinical care. Specific subject inclusion and exclusion criteria are included below.

Subjects met all the following inclusion criteria to be enrolled in the study:

- 18 years of age or older
- Presentation with a clinical condition characterized by neurological signs and symptoms that, in the opinion of the investigator, include embolic stroke or transient ischemic attack in the differential diagnosis
- Scheduled for TTE study with agitated saline contrast (bubble study) within ±30 days of informed consent
- Ability to successfully perform a Valsalva maneuver.
- Signed informed consent
- Ability to comply with the protocol

Subjects were not enrolled in the study if any of the exclusion criteria were met:

- History of RLS/PFO closure
- Pregnancy or lactation at the time of study
- History of partial or full craniotomy/craniectomy within the past 6 months
- Presence of a physical limitation preventing TCD/Headmount placement

### Trial Procedures

Enrolled patients underwent raTCD (NovaGuide Intelligent Ultrasound, NeuraSignal Inc, Los Angeles, CA) in addition to standard of care TTE with agitated saline contrast bubble study, both within 30 days of informed consent. The raTCD is a five-degree-of-freedom robotic unit paired with a signal optimization algorithm that supports traditional 2 MHz diagnostic TCD. Any other diagnostics for RLS testing (eg, TEE or TCD) were optional, performed only at the discretion of the treatment team. A clinical research coordinator performed the raTCD procedure. The research coordinators had no prior TCD experience before being trained to perform standard TCD bubble study technique^19^ and the study protocol for raTCD RLS testing. Injections of agitated saline contrast during raTCD were performed at rest and with calibrated Valsalva (mean flow velocity drop of at least 25% and obvious characteristic waveform changes), both in supine and elevated (45°) positions by the clinical research coordinator or bedside nurse. TTE raw data (still and video) were interpreted locally and reviewed in a cardiology core laboratory by independent, blinded experts. With the intent of having standard of care TTE as the control, TTE performance was not standardized but rather performed in accordance with the established clinical protocol of the local study site. The raTCD studies, including 60-second audio/video captures of the raw data, were interpreted in a TCD Core Lab by independent, blinded experts. RLS presence and size on raTCD were graded by Spencer Logarithmic Scale criteria^16^ and International Consensus Criteria.^19^ For the purpose of prespecified secondary outcome analysis,^23^ large RLS was defined by >20 bubbles in the left heart on TTE^25^ and Spencer Logarithmic Scale grade 3 or higher on raTCD.^18^ Further details on the trial procedures are available in the Supplemental Appendix.

### Outcomes

The primary outcome was rate of RLS detection with TTE and raTCD in the intent to scan (ITS) cohort, which included site assessment of TTE (eg, local clinical interpretation). The primary safety outcome was any serious device-related adverse events. The key prespecified secondary outcome was rate of detection of large RLS on TTE and raTCD. The other prespecified secondary outcomes, including rate of absence of transtemporal windows and device performance parameters, are in the Supplemental Appendix.

### Statistical Analysis

The study was powered based on the results of a meta-analysis^13^ reporting a pooled TCD sensitivity of 96.1% for PFO detection, while the pooled TTE sensitivity was estimated at 45.1% (absolute difference of 51%). For power calculations, we used a more moderate effect size of 40% increase in the sensitivity of raTCD TTE. A sample size of 100 subjects achieves 90% power to detect a difference of 40% between the 2 diagnostic tests whose sensitivities are 90% (TCD) and 50% (TTE). This procedure uses a 2-sided McNemar test with a significance level of 0.05. The mean prevalence of PFO in the population of patients with cryptogenic stroke was estimated to be at least 30%.^26^ The proportion of discordant pairs has been set at 0.500. Given previous reports^27–29^ indicating a prevalence of suboptimal transtemporal windows in 5% of Hispanic, 5% of White, 9% in African American, and 14% of Asian individuals, we increased our projected sample size by 20% (n=120). In addition, the final sample size was further increased to account for an anticipated dropout rate of at least 20%. Consequently, the study sample was set to at least 150 individuals. Data were analyzed on an ITS and per protocol basis. The ITS cohort was defined as subjects that met all inclusion/exclusion criteria and raTCD was attempted. The per protocol cohort was defined as subjects that met all inclusion/exclusion criteria, successfully completed the study with no protocol deviations and had complete data sets. Any data loss of the raTCD or TTE was treated as a dropout. We presented continuous parametric data using their mean values together with their corresponding SDs. We used median values for the presentation of nonparametric data and percentages for all dichotomous variables. Statistical comparisons between different subgroups were performed using the unpaired *t* test and Mann-Whitney *U* test as appropriate.

## RESULTS

### Characteristics of the Patients

**Figure 1.**
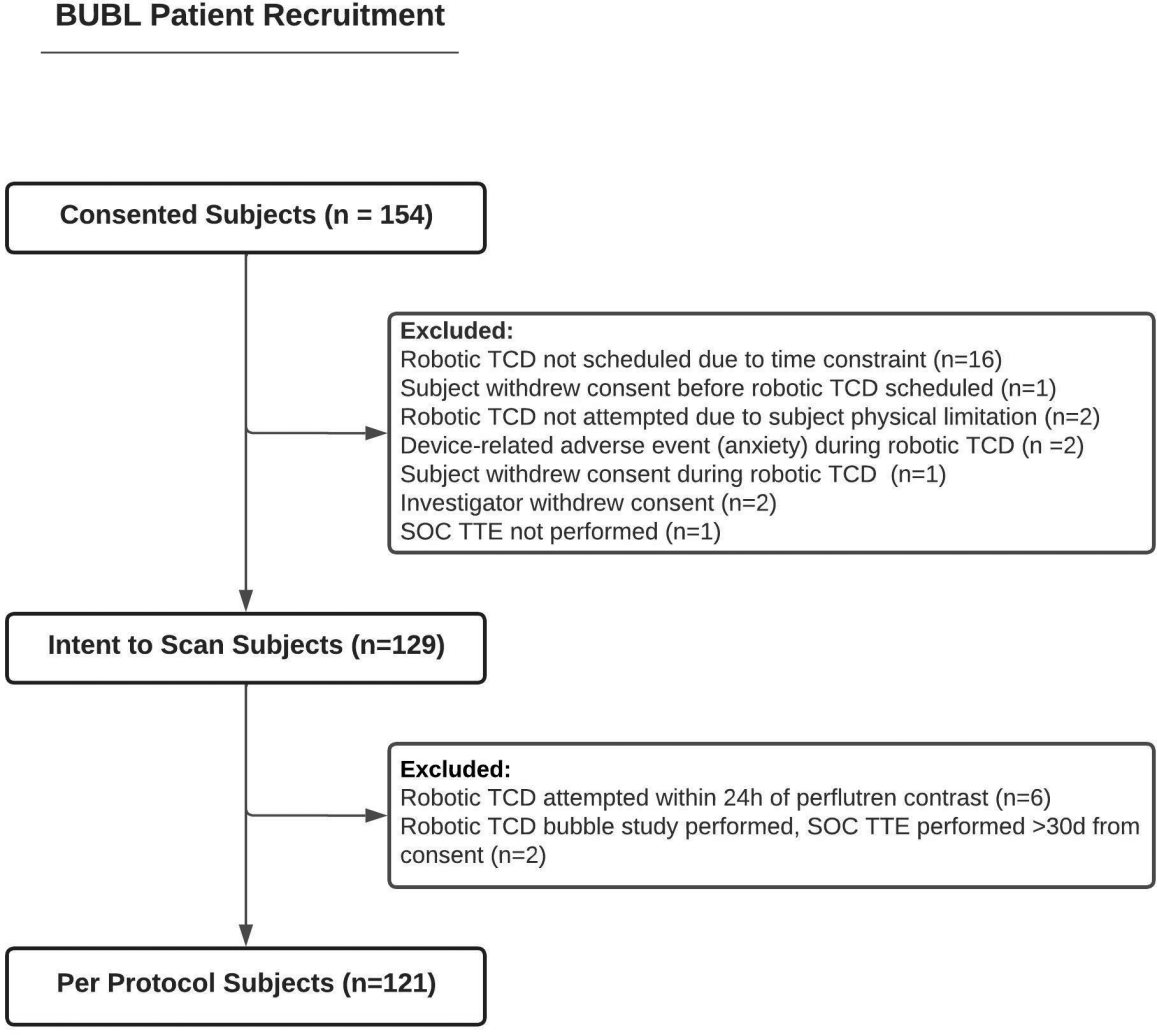
**BUBL patient recruitment diagram.** SOC indicates standard of care; TCD, transcranial Doppler; and TTE, transthoracic echocardiography.

From October 6, 2020 to October 20, 2021, a total of 154 patients were consented and enrolled, of whom 129 were evaluated on an ITS basis (Figure 1), and 121 subjects were evaluable as the per protocol cohort. Sixty one (47%) subjects were women and the mean age was 60±15 years in the ITS cohort. The qualifying clinical event was an acute ischemic stroke or transient ischemic attack in all subjects, with a majority (73; 57%) diagnosed with an embolic stroke of undetermined source. The no window rate in the ITS population was 7%. Baseline characteristics of the patients are presented in Table 1.

**Table 1.**
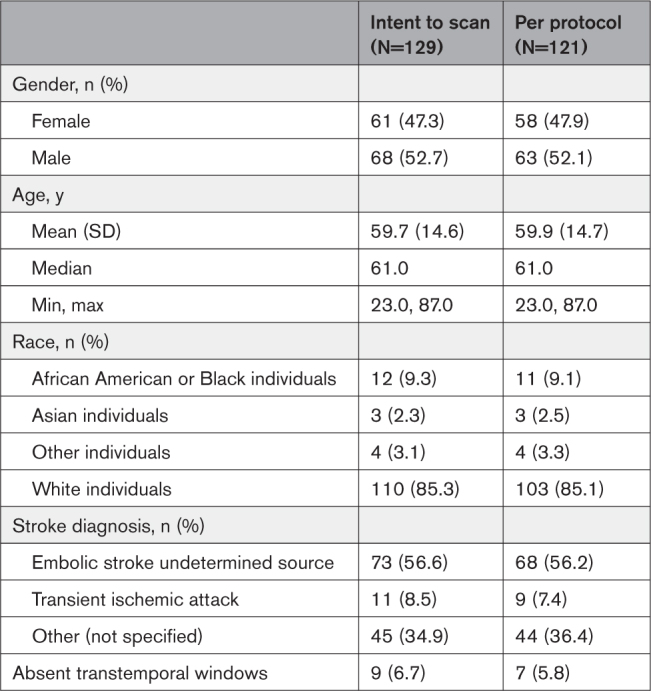
Demographics of the Study Population

### Outcomes

For the primary outcome, in the ITS cohort, raTCD detected any RLS in 82 patients (64%) whereas TTE documented any RLS in 26 (20%) patients (absolute difference 43.4% [95% CI, 35.2%–52.0%]; *P*<0.001; Table 2). The per protocol analysis of this same end point is included in Table 2.

**Table 2.**
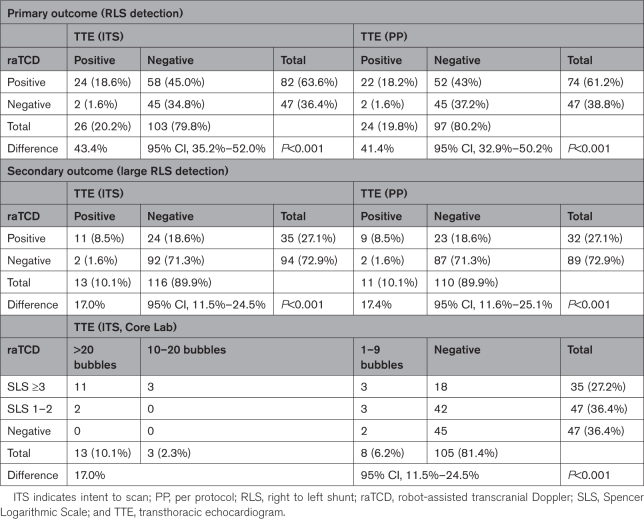
Outcomes

For the secondary prespecified outcome of large RLS detection, raTCD detected large RLS in 35 patients (27%) and TTE found large RLS in 13 (10%; absolute difference 17.0% [95% CI, 11.5%–24.5%]; *P*<0.001). TTE showed no RLS in 18 of 35 (51%) large RLS diagnosed by raTCD (Table 2).

There were few TCD and TEE data, which were optional diagnostics in this study. Overall, there were 14 cases with TEE (11%) and 6 of those also had evaluable TCD (5%) for cross-comparison (Table 3). Prespecified secondary analyses related to TEE and TCD included percent detection and are included in Table 4. There was a significant difference in percent detection of RLS between raTCD and both TCD (86% versus 57%; *P*=0.041) and TEE Core Lab analysis (86% versus 43%; *P*=0.041).

**Table 3.**
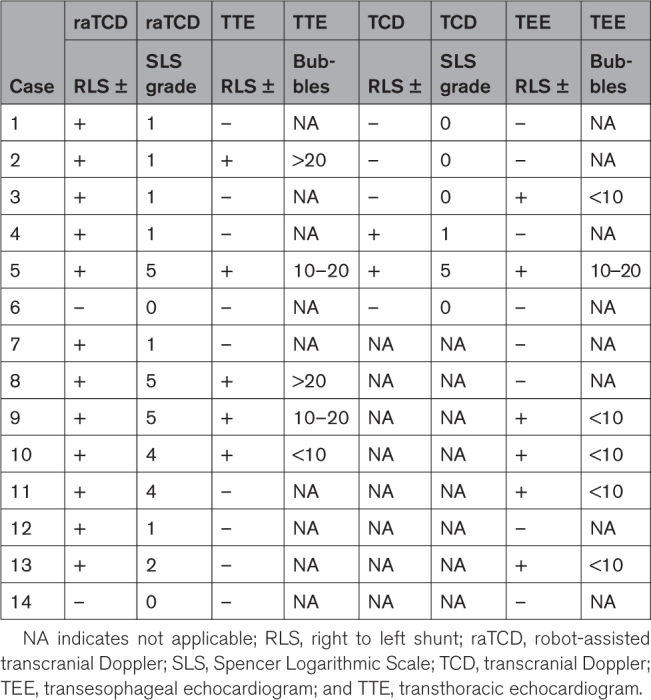
Outcomes in Subjects With TEE

**Table 4.**
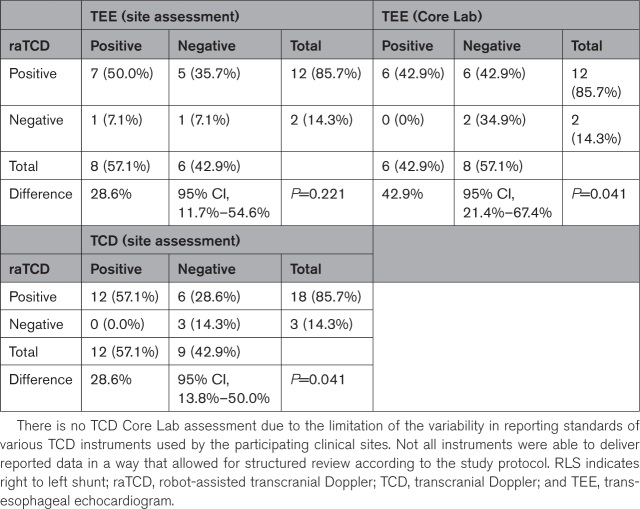
RLS% Detection raTCD Versus TEE and TCD

### Safety

There were no serious adverse events in this study related to the device or microbubble contrast injection. There were 2 nonserious adverse events (anxiety) in the ITS population.

## DISCUSSION

**Figure 2.**
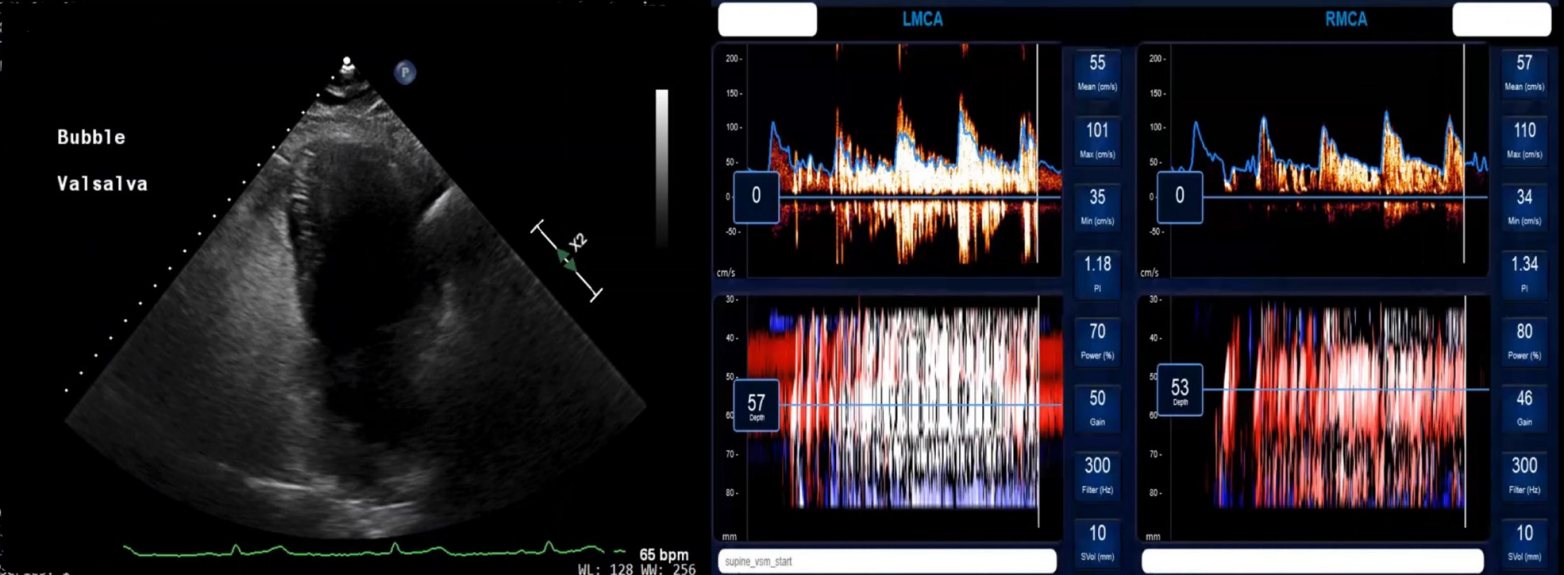
**A transthoracic echocardiogram (TTE) and robot-assisted transcranial Doppler (raTCD) in one of the study subjects with a negative TTE and a strongly positive raTCD.** The 4-chamber apical view of the TTE demonstrates opacification of the right atrium and ventricle (on the **left** of the figure) with agitated saline contrast and none of those bubbles crossing into the left atrium or ventricle, suggesting the absence of a right to left shunt (RLS). In stark contrast, one can see on the **right** part of the figure that many bubbles, generating the white streaks seen on the raTCD—enough to fill entire cardiac cycles—indicating a large RLS. In the setting of discrepancy, the positive raTCD study is to be trusted as TTE is subject to false negative and the pattern of the result of raTCD does not suggest false positive. Please see Video S2 for audio and visual detail.

This study is the first multicenter, prospective study of the diagnostic accuracy, feasibility, and safety of raTCD as compared with standard of care TTE for the diagnosis of RLS. In this study, raTCD was ≈3 times more likely to diagnose any RLS presence as compared with TTE. It was also safe and technically feasible to obtain quality raTCD results with an operator who had no prior TCD skills. Importantly, raTCD detected large^18^ RLS at ≈3 times the rate of TTE. Otherwise stated, TTE completely missed or underdiagnosed approximately two thirds of the large RLS diagnosed by raTCD (Figure 2). Considering TTE is the most common screening diagnostic for RLS, our results suggest RLS are frequently underdiagnosed. The fact that TTE showed no signs of any RLS in half of the large RLS diagnosed by raTCD should be a signal for change in practice to those caring for stroke patients. These data are only the most recent in a long line of observational studies over the last 3 decades noting a remarkably consistent outcome of TCD being more sensitive to detect RLS and PFO than TTE. While TTE is of use in the workup of embolic stroke because it provides diagnostic information other than RLS status, TCD—standard or robot-assisted—may be considered as the front-line screening examination for RLS rather than TTE.

Our results comport with prior studies. TCD has a long, globally published experience as a highly sensitive diagnostic for RLS detection,^18,30–37^ and has been previously compared with TTE and TEE in smaller series^38–40^ of varying study design, with consistently favorable (>90%) sensitivity as compared with the gold standard of TEE. For the specific diagnosis of PFO, 2 meta-analyses^13,14^ have demonstrated an ≈40% difference in PFO rate of detection between TCD and TTE, with TCD demonstrating ≈95% sensitivity and TTE ≈45% sensitivity overall as compared with TEE. Our finding of an absolute difference of 43% in RLS detection between raTCD and TTE, collected prospectively in a multicenter device trial format, aligns with these previous estimations and lends credence to their veracity. Our results suggest that an autonomous, five-degree-of-freedom robot with machine-learning-supported signal location and optimization algorithms reinforcing standard TCD instrumentation can achieve this known sensitivity of TCD for the diagnosis of RLS without the need for expert practitioners, who are scarce, to perform the test. We also provide reassuring data regarding the safety of raTCD with agitated saline injection, consistent with previous reports of the excellent safety profile of agitated saline contrast TCD bubble studies for RLS detection.^41^

As previously mentioned, prior studies estimate RLS prevalence in a patient population similar to ours to be as high as 50%.^1–4^ However, we found a higher percentage of 63.6%. These prior series were based on echocardiographic data. In light of our findings, we conclude that the higher than expected detection rate is not only from subject selection focused on patients with probable cerebral embolic events, which was a feature of these previous studies, but also the known ability of TCD to detect the presence of small and extracardiac shunts with greater sensitivity than echocardiography.

Our study has limitations. Our population was predominantly older and white and, considering transtemporal window adequacy varies with age, sex, and ethnicity, a more diverse study population may have yielded a different no windows rate, but this limitation is unlikely to have affected the primary outcome. We cannot comment definitively on accuracy parameters of raTCD versus TEE or standard TCD testing because very few patients had all optional diagnostic tests performed. That said, as outlined in Tables 3 and 4, there is a signal of accuracy that is in line with the aforementioned studies and routine clinical practice: raTCD was positive for RLS in all cases TEE was positive, there were no cases of TEE positive and raTCD negative for RLS (eg, “false negative”), and all of the “false positive” raTCD results were Spencer Logarithmic Scale grade 1, indicating a small shunt that could conceivably be beneath the resolution of TEE. There was, per se, a significant difference (*P*=0.041) between raTCD and both TEE (Core Lab analysis) and TCD for the percent detection of RLS favoring raTCD.

Another limitation of this study is that the technique for the standard of care TTE bubble study was not specified in the methods, whereas the TCD bubble study followed a study-defined protocol. We acknowledge that factors such as the quality of injection, patient positioning, Valsalva technique, and bubble contrast preparation may have introduced bias in favor of raTCD into these results, but the hope of this approach was to have true standard of care as the control of this study. In addition, participating sites included high-volume comprehensive stroke centers and the predominantly acute ischemic stroke/transient ischemic attack patient sample may not represent the exact clinical population seen at an outpatient neurology clinic or echocardiographic practice. However, our study provides data for a common clinical scenario where accurate RLS detection is paramount.

These findings have a mixed standing when contextualized within current guidelines on the use of TCD for RLS evaluation. A European multisociety-supported position article on the management of patients with PFO states “[contrast]-TCD has a higher sensitivity than [contrast]-TTE as a first-line investigation to detect a right to left shunt,” granting a level of evidence “A” and a “conditional” strength of recommendation. The diagram of recommended diagnostics for RLS detection aligns with our findings, namely that TCD is a reasonable front-line screen and, if TTE is negative, to proceed to TCD given the superior sensitivity and concordance with TEE.^42^ In the most recent guideline from the American Heart Association/American Stroke Association (AHA/ASA) for the prevention of stroke in patients with stroke and transient ischemic attack, TCD is given a level of evidence C-LD (limited data), class of recommendation 2b (weak) for use “[i]n patients with ischemic stroke or transient ischemic attack in whom PFO closure would be contemplated…” It is not mentioned in the subsection on PFO nor does it appear in any of the flowcharts of recommended diagnostic studies in spite of acknowledging “TCD compares favorably with TEE for detecting right-to-left shunting…”^43^ Furthermore, according to the American Academy of Neurology Practice Advisory Update on PFO and secondary stroke prevention, “…TCD has been demonstrated to have similar sensitivity and specificity to TEE to detect right to left shunting…” and “in patients being considered for PFO closure, clinicians may use TCD with agitated saline contrast as a screening evaluation for right to left shunt.”^44^ Our data support a revisitation of the AHA/ASA and American Academy of Neurology guidelines and recommended diagnostics for RLS detection and secondary stroke prevention given that the currently recommended workflow predicated on TTE as a screening examination and a focus on PFO closure alone rather than considering the multiple mechanisms by which RLS can present stroke risk, surely leads to significant underdiagnosis of RLS based on our findings, and thereby missed opportunities to effectively prevent stroke recurrence.

In conclusion, raTCD was ≈3 times more likely to diagnose any RLS, including large PFO, in this cohort of patients as compared with TTE. TTE failed to diagnose approximately two thirds of the large RLS diagnosed by raTCD. These findings, buttressed by the aforementioned global, decades-long experience with TCD for RLS testing, support TCD as the superior initial screening test for RLS as compared with TTE. The raTCD was safe and feasible for use by personnel without TCD expertise, suggesting that raTCD can achieve the known sensitivity of TCD for RLS without an experienced operator to perform the examination.

## ARTICLE INFORMATION

### Sources of Funding

This work was funded by NeuraSignal, Inc.

### Disclosures

Drs Alexandrov, Rubin, Tsivgoulis, and Volpi have received payment as consultants to the study sponsor. C. Douville has received travel support from the study sponsor. The other authors report no conflicts.

### Supplemental Material

Trial Protocol

Statistical Analysis Plan

Supplemental Appendix

Investigators

Supplemental Methods

Tables S1–S4

Figure S1

Video S1

STARD Checklist

CONSORT Checklist

## Supplementary Material



## References

[1] Saver JL. Cryptogenic stroke. N Engl J Med. 2016;374:2065–2074. doi: 10.1056/NEJMcp1503946 27223148 10.1056/NEJMcp1503946

[2] Hagen PT Scholz DG Edwards WD. Incidence and size of patent foramen ovale during the first 10 decades of life: an autopsy study of 965 normal hearts. Mayo Clin Proc. 1984;59:17–20. doi: 10.1016/s0025-6196(12)60336-x 6694427 10.1016/s0025-6196(12)60336-x

[3] Lechat P Mas JL Lascault G Loron P Theard M Klimczac M Drobinski G Thomas D Grosgogeat Y. Prevalence of patent foramen ovale in patients with stroke. N Engl J Med. 1988;318:1148–1152. doi: 10.1056/NEJM198805053181802 3362165 10.1056/NEJM198805053181802

[4] Elgendy AY Saver JL Amin Z Boudoulas K Carroll J Elgendy IY Grunwald IQ Gertz ZM Hijazi ZM Horlick EM. Proposal for updated nomenclature and classification of potential causative mechanism in patent foramen ovale-associated stroke. JAMA Neurol. 2020;77:878–886. doi: 10.1001/jamaneurol.2020.0458 32282016 10.1001/jamaneurol.2020.0458

[5] Mas JL Arquizan C Lamy C Zuber M Cabanes L Derumeaux G Coste J; Patent Foramen Ovale and Atrial Septal Aneurysm Study Group. Recurrent cerebrovascular events associated with patent foramen ovale, atrial septal aneurysm, or both. N Engl J Med. 2001;345:1740–1746. doi: 10.1056/NEJMoa011503 11742048 10.1056/NEJMoa011503

[6] Kent DM Ruthazer R Weimar C Mas JL Serena J Homma S Di Angelantonio E Di Tullio MR Lutz J Elkind MSV. An index to identify stroke-related vs incidental patent foramen ovale in cryptogenic stroke. Neurology. 2013;81:619–625. doi: 10.1212/WNL.0b013e3182a08d59 23864310 10.1212/WNL.0b013e3182a08d59 PMC3775694

[7] Mas JL Derumeaux G Guillon B Massardier E Hosseini H Mechtouff L Arquizan C Bejot Y Vuillier F Detante O; CLOSE Investigators. Patent foramen ovale closure or anticoagulation vs antiplatelets after stroke. N Engl J Med. 2017;377:1011–1021. doi: 10.1056/NEJMoa1705915 28902593 10.1056/NEJMoa1705915

[8] Søndergaard L Kasner SE Rhodes JF Andersen G Iversen H Nielsen-Kudsk JE Settergren M Sjostrand C Roine RO Hildick-Smith D. Patent foramen ovale closure or antiplatelet therapy for cryptogenic stroke. N Engl J Med. 2017;377:1033–1042. doi: 10.1056/NEJMoa1707404 28902580 10.1056/NEJMoa1707404

[9] Saver JL Carroll JD Thaler DE Smalling RW MacDonald LA Marks DS Tirschwell DL; RESPECT Investigators. Long-term outcomes of patent foramen ovale closure or medical therapy after stroke. N Engl J Med. 2017;377:1022–1032. doi: 10.1056/NEJMoa1610057 28902590 10.1056/NEJMoa1610057

[10] Lee PH Song JK Kim JS Heo R Lee S Kim DH Song JM Kang DH Kwon SU Kang DW. Cryptogenic stroke and high-risk patent foramen ovale: the DEFENSE-PFO trial. J Am Coll Cardiol. 2018;71:2335–2342. doi: 10.1016/j.jacc.2018.02.046 29544871 10.1016/j.jacc.2018.02.046

[11] Turc G Calvet D Guérin P Sroussi M Chatellier G Mas JL; CLOSE Investigators. Closure, anticoagulation, or antiplatelet therapy for cryptogenic stroke with patent foramen ovale: systematic review of randomized trials, sequential meta-analysis, and new insights from the CLOSE study. J Am Heart Assoc. 2018;7:e008356. doi: 10.1161/JAHA.117.008356 29910193 10.1161/JAHA.117.008356 PMC6220551

[12] Tsivgoulis G Katsanos AH Mavridis D Frogoudaki A Vrettou AR Ikonomidis I Parissis J Deftereos S Karapanayiotides T Palaiodimou L. Percutaneous patent foramen ovale closure for secondary stroke prevention: network meta-analysis. Neurology. 2018;91:e8–e18. doi: 10.1212/WNL.0000000000005739 29875217 10.1212/WNL.0000000000005739

[13] Katsanos AH Psaltopoulou T Sergentanis TN Frogoudaki A Vrettou AR Ikonomidis I Paraskevaidis I Parissis J Bogiatzi C Zompola C. Transcranial Doppler versus transthoracic echocardiography for the detection of patent foramen ovale in patients with cryptogenic cerebral ischemia: a systematic review and diagnostic test accuracy meta-analysis: TCD vs TTE for PFO. Ann Neurol. 2016;79:625–635. doi: 10.1002/ana.24609 26833864 10.1002/ana.24609

[14] Mojadidi MK Winoker JS Roberts SC Msaouel P Zaman MO Gevorgyan R Tobis JM. Accuracy of conventional transthoracic echocardiography for the diagnosis of intracardiac right-to-left shunt: a meta-analysis of prospective studies. Echocardiogr. 2014;31:1036–1048. doi: 10.1111/echo.12583 10.1111/echo.12583 24689727

[15] Hahn RT Abraham T Adams MS Bruce CJ Glas KE Lang RM Reeves ST Shanewise JS Siu SC Stewart W. Guidelines for performing a comprehensive transesophageal echocardiographic examination: recommendations from the American society of echocardiography and the society of cardiovascular anesthesiologists. J Am Soc Echocardiogr. 2013;26:921–964. doi: 10.1016/j.echo.2013.07.009 23998692 10.1016/j.echo.2013.07.009

[16] Spencer MP Moehring MA Jesurum J Gray WA Olsen JV Reisman M. Power M-mode transcranial Doppler for diagnosis of patent foramen ovale and assessing transcatheter closure. J Neuroimaging. 2004;14:342–349. doi: 10.1177/1051228404268743 15358955 10.1177/1051228404268743

[17] Lao AY Sharma VK Tsivgoulis G Malkoff MD Alexandrov AV Frey JL. Effect of body positioning during transcranial Doppler detection of right-to-left shunts. Eur J Neurol. 2007;14:1035–1039. doi: 10.1111/j.1468-1331.2007.01879.x 17718697 10.1111/j.1468-1331.2007.01879.x

[18] Lao AY Sharma VK Tsivgoulis G Frey JL Malkoff MD Navarro JC Alexandrov AV. Detection of right-to-left shunts: comparison between the international consensus and spencer logarithmic scale criteria. J Neuroimaging Off J Am Soc Neuroimaging. 2008;18:402–406. doi: 10.1111/j.1552-6569.2007.00218.x 10.1111/j.1552-6569.2007.00218.x 18333839

[19] Jauss M Zanette E. Detection of right-to-left shunt with ultrasound contrast agent and transcranial Doppler sonography. Cerebrovasc Dis. 2000;10:490–496. doi: 10.1159/000016119 11070388 10.1159/000016119

[20] Alexandrov AV Sloan MA Tegeler CH Newell DN Lumsden A Garami Z Levy C Wong LKS Douville C Kaps M. Practice standards for transcranial Doppler (TCD) ultrasound. Part II. Clinical indications and expected outcomes. J Neuroimaging Off J Am Soc Neuroimaging. 2012;22:215–224. doi: 10.1111/j.1552-6569.2010.00523.x 10.1111/j.1552-6569.2010.00523.x 20977531

[21] Khan DZ Placek MM Smielewski P Budohoski KP Anwar F Hutchinson PJA Bance M Czosnyka M Helmy A. Robotic semi-automated transcranial Doppler assessment of cerebrovascular autoregulation in post-concussion syndrome: methodological considerations. Neurotrauma Rep. 2020;1:218–231. doi: 10.1089/neur.2020.0021 33274347 10.1089/neur.2020.0021 PMC7703686

[22] Clare K Stein A Damodara N Feldstein E Alshammari H Ali S Kurian C Rosenberg J Bauerschmidt A Kaur G. Safety and efficacy of a novel robotic transcranial Doppler system in subarachnoid hemorrhage. Sci Rep. 2022;12:2266. doi: 10.1038/s41598-021-04751-1 35145104 10.1038/s41598-021-04751-1 PMC8831519

[23] Rubin MN Alexandrov AV Douville C Rinsky B Tsivgoulis G. Novel robotic TCD ultrasound with bubbles versus standard care to detect right to left shunt: study methods. J Neuroimaging Off J Am Soc Neuroimaging. 2021;31:858–863. doi: 10.1111/jon.12890 10.1111/jon.12890 PMC8518840 34081363

[24] Bossuyt PM Reitsma JB Bruns DE Gatsonis CA Glasziou PP Irwig L Lijmer JG Moher D Rennie D de Vet HCW; STARD Group. for the STARD Group STARD 2015: An updated list of essential items for reporting diagnostic accuracy studies. BMJ. 2015;351:h5527. doi: 10.1136/bmj.h5527 26511519 10.1136/bmj.h5527 PMC4623764

[25] Saric M Armour AC Arnaout MS Chaudhry FA Grimm RA Kronzon I Landeck BF Maganti K Michelena HI Tolstrup K. Guidelines for the use of echocardiography in the evaluation of a cardiac source of embolism. J Am Soc Echocardiogr. 2016;29:1–42. doi: 10.1016/j.echo.2015.09.011 26765302 10.1016/j.echo.2015.09.011

[26] Katsanos AH Giannopoulos S Frogoudaki A Vrettou AR Ikonomidis I Paraskevaidis I Zompola C Vadikolias K Boviatsis E Parissis J. The diagnostic yield of transesophageal echocardiography in patients with cryptogenic cerebral ischaemia: a meta-analysis. Eur J Neurol. 2016;23:569–579. doi: 10.1111/ene.12897 26918744 10.1111/ene.12897

[27] Itoh T Matsumoto M Handa N Maeda H Hougaku H Hashimoto H Etani H Tsukamoto Y Kamada T. Rate of successful recording of blood flow signals in the middle cerebral artery using transcranial Doppler sonography. Stroke. 1993;24:1192–1195. doi: 10.1161/01.str.24.8.1192 8342196 10.1161/01.str.24.8.1192

[28] Marinoni M Ginanneschi A Forleo P Amaducci L. Technical limits in transcranial Doppler recording: inadequate acoustic windows. Ultrasound Med Biol. 1997;23:1275–1277. doi: 10.1016/s0301-5629(97)00077-x 9372576 10.1016/s0301-5629(97)00077-x

[29] Brunser AM Silva C Cárcamo D Munoz P Hoppe A Olavarria VV Diaz V Abarca J. Transcranial Doppler in a Hispanic-Mestizo population with neurological diseases: a study of sonographic window and its determinants. Brain Behav. 2012;2:231–236. doi: 10.1002/brb3.46 22741096 10.1002/brb3.46 PMC3381627

[30] Chimowitz MI Nemec JJ Marwick TH Lorig RJ Furlan AJ Salcedo EE. Transcranial Doppler ultrasound identifies patients with right-to-left cardiac or pulmonary shunts. Neurology. 1991;41:1902–1904. doi: 10.1212/wnl.41.12.1902 1745345 10.1212/wnl.41.12.1902

[31] Teague SM Sharma MK. Detection of paradoxical cerebral echo contrast embolization by transcranial Doppler ultrasound. Stroke. 1991;22:740–745. doi: 10.1161/01.str.22.6.740 2057972 10.1161/01.str.22.6.740

[32] Di Tullio M Sacco RL Venketasubramanian N Sherman D Mohr JP Homma S. Comparison of diagnostic techniques for the detection of a patent foramen ovale in stroke patients. Stroke. 1993;24:1020–1024. doi: 10.1161/01.str.24.7.1020 8322376 10.1161/01.str.24.7.1020

[33] Anzola GP Renaldini E Magoni M Costa A Cobelli M Guindani M. Validation of transcranial doppler sonography in the assessment of patent foramen ovale. Cerebrovasc Dis. 1995;5:194–198. doi: 10.1159/000107851

[34] Zanette EM Mancini G De Castro S Solaro M Cartoni D Chiarotti F. Patent foramen ovale and transcranial Doppler comparison of different procedures. Stroke. 1996;27:2251–2255. doi: 10.1161/01.str.27.12.2251 8969789 10.1161/01.str.27.12.2251

[35] Devuyst G Despland PA Bogousslavsky J Jeanrenaud X. Complementarity of contrast transcranial Doppler and contrast transesophageal echocardiography for the detection of patent foramen ovale in stroke patients. Eur Neurol. 1997;38:21–25. doi: 10.1159/000112897 10.1159/000112897 9252794

[36] Droste DW Reisener M Kemény V Dittrich R Schulte-Altedorne G Stypmann J Wichter T Ringelstein EB. Contrast transcranial Doppler ultrasound in the detection of right-to-left shunts reproducibility, comparison of 2 agents, and distribution of microemboli. Stroke. 1999;30:1014–1018. doi: 10.1161/01.str.30.5.1014 10229737 10.1161/01.str.30.5.1014

[37] Tobe J Bogiatzi C Munoz C Tamayo A Spence JD. Transcranial Doppler is complementary to echocardiography for detection and risk stratification of patent foramen ovale. Can J Cardiol. 2016;32:986.e9–986.e16. doi: 10.1016/j.cjca.2015.12.009 10.1016/j.cjca.2015.12.009 26952158

[38] Nemec JJ Marwick TH Lorig RJ Davison MB Chimowitz MI Litowitz H Salcedo EE. Comparison of transcranial Doppler ultrasound and transesophageal contrast echocardiography in the detection of interatrial right-to-left shunts. Am J Cardiol. 1991;68:1498–1502. doi: 10.1016/0002-9149(91)90285-s 1746433 10.1016/0002-9149(91)90285-s

[39] Caputi L Carriero MR Falcone C Parati E Piotti P Materazzo C Anzola GP. Transcranial Doppler and transesophageal echocardiography: comparison of both techniques and prospective clinical relevance of transcranial Doppler in patent foramen ovale detection. J Stroke Cerebrovasc Dis Off J Natl Stroke Assoc. 2009;18:343–348. doi: 10.1016/j.jstrokecerebrovasdis.2008.12.001 10.1016/j.jstrokecerebrovasdis.2008.12.001 19717016

[40] Mahmoud AN Elgendy IY Agarwal N Tobis JM Mojadidi MK. Identification and quantification of patent foramen ovale-mediated shunts: echocardiography and transcranial Doppler. Interv Cardiol Clin. 2017;6:495–504. doi: 10.1016/j.iccl.2017.05.002 28886841 10.1016/j.iccl.2017.05.002

[41] Tsivgoulis G Stamboulis E Sharma VK Heliopoulos I Voumvourakis K Teoh HL Vadikolias K Triantafyllou N Chan BPL Vasdekis SN. Safety of transcranial Doppler “bubble study” for identification of right to left shunts: an international multicentre study. J Neurol Neurosurg Psychiatry. 2011;82:1206–1208. doi: 10.1136/jnnp.2010.219733 20971751 10.1136/jnnp.2010.219733

[42] Pristipino C Sievert H D’Ascenzo F Mas JL Meier B Scacciatella P Hildick-Smith D Gaita F Toni D Kyrle P; European Association of Percutaneous Cardiovascular Interventions (EAPCI). European position paper on the management of patients with patent foramen ovale general approach and left circulation thromboembolism. EuroIntervention. 2019;14:1389–1402. doi: 10.4244/EIJ-D-18-00622 30141306 10.4244/EIJ-D-18-00622

[43] Kleindorfer DO Towfighi A Chaturvedi S Cockroft KM Gutierrez J Lombardi-Hill D Kamel H Kernan WN Kittner SJ Leira EC. 2021 Guideline for the prevention of stroke in patients with stroke and transient ischemic attack: a guideline from the American heart association/American stroke association. Stroke. 2021;52:e364–e467. doi: 10.1161/STR.0000000000000375 34024117 10.1161/STR.0000000000000375

[44] Messé SR Gronseth GS Kent DM Kizer JR Homma S Rosterman L Carroll JD Ishida K Sangha N Kasner SE. Practice advisory update summary: patent foramen ovale and secondary stroke prevention: report of the guideline subcommittee of the American academy of neurology. Neurology. 2020;94:876–885. doi: 10.1212/WNL.0000000000009443 32350058 10.1212/WNL.0000000000009443 PMC7526671

