# Supplementary material for: Robot-Assisted Transcranial Doppler Versus Transthoracic Echocardiography for Right to Left Shunt Detection
Source: Stroke. 2023 Oct 5;54(11):2842–50. doi: 10.1161/STROKEAHA.123.043380 (PMC10589435; doi:10.1161/STROKEAHA.123.043380)
Supplement: Supplementary file 1 [file str-54-2842-s001.pdf]

RoBotic TCD Ultrasound BubbLe Study Compared to Transthoracic Echocardiography for Detection of Right to Left Shunt

The BUBL Study

Supplementary Appendix

Contents

Investigators 2
University of Tennessee Health Science Center 2
CHI Memorial Hospital 2
Barrow Neurological Institute 2
Swedish Medical Center 2
Houston Methodist 2
Providence 2
Sponsor 2
Methods 2
Inclusion/Exclusion Criteria 2
Trial Protocol 2
Trial Procedures 2
Definition of “Large RLS” 3
Figures & Tables 3
Figures 3
Figure 1: NeuralBot Investigational System, raTCD 3
Supplemental Video: TTE negative, raTCD positive for large RLS 4
Tables 5
Table 1 Subjects Enrolled by Study Site 5
Table 2.1 Imaging Accountability 5
Table 2.2: Outcomes in subjects with TEE 6
Table 2.3 RLS detection accuracy: raTCD vs TEE 6
Table 2.4 RLS detection accuracy: TTE vs TEE 6
Table 2.5 RLS detection accuracy: raTCD vs TTE 7
Table 2.6 RLS % detection: raTCD vs TEE 7
Table 2.7 RLS % detection: raTCD vs TCD 7
Table 3: Transtemporal window(s) 7
Table 4: Success rate of raTCD 7

Investigators

University of Tennessee Health Science Center

- National Principal Investigator: M. Rubin, MD
- Steering Committee Chair: A. Alexandrov, MD

CHI Memorial Hospital

- Site Principal Investigator: R. Shah, MD
- Other Investigators: Kim Gray, Rafael Souchet, ANP, Sara-Page Red, ANP, Tracy Robertson, ANP

Barrow Neurological Institute

- Site Principal Investigator: T. Youn, MD

Swedish Medical Center

- Site Principal Investigator: A. Stayman, MD

Houston Methodist

- Site Principal Investigator: J. Volpi, MD
- Other Investigators: Jonathan Wiese, MSN, NP, Amber Criswell, CCRC

Providence

- Site Principal Investigator: T. Lowenkopf, MD

Sponsor

- Robert Hamilton, PhD
- Dave Juco
- Lucy Reynell

Methods

Inclusion/Exclusion Criteria

Subjects met all the following inclusion criteria to be enrolled in the study:

- 18 years of age or older.
- presentation with a clinical condition characterized by neurological signs and symptoms that, in the opinion of the investigator, include embolic stroke or TIA in the differential diagnosis.
- scheduled for TTE study with agitated saline contrast (bubble study) within ±30 days of informed consent.
- ability to successfully perform a Valsalva Maneuver (VM).
- signed informed consent
- ability to comply with the protocol.

Subjects were not enrolled in the study if any of the following exclusion criteria were met:

- history of RLS/PFO closure.
- pregnancy or lactation at time of study
- history of partial or full craniotomy/craniectomy within the past 6 months.
- presence of a physical limitation preventing TCD/Headmount placement.

Trial Protocol

The trial protocol was submitted in its entirety for editorial review and is available for download.

Trial Procedures

See section 6 in the trial protocol for all details of trial procedures.

Definition of "Large RLS"

The consensus definition of large, potentially intervenable shunt used routinely in clinical practice is that which conveys at least 30 bubbles noted in bilateral monitoring, corresponding to a grade 3 in both the Spencer Logarithmic Scale and International Consensus Criteria. (Lao, et al 2008, PMID: 18333839)

There is no obvious consensus of large, potentially intervenable shunt as defined by bubble count in echocardiography guidelines or clinical trials. The clinical trials establishing patent foramen ovale closure with antiplatelet as superior to antiplatelet alone for stroke risk reduction in select patients variably defined "large" PFO as 25-30 microbubbles. An American Society of Echocardiography Guideline (Saric, et al 2016, PMID 26765302) suggests >20 bubbles in the left heart on TTE as a large shunt. The investigators elected to use the most inclusive criterion of >20 bubbles on TTE for this study.

Figures & Tables

Figures

Figure 1: NeuralBot Investigational System, raTCD

The NeuralBot Investigational System, or robot-assisted TCD (raTCD), is comprised of both the Lucid M1 System and the NeuralBot accessory. The NeuralBot accessory must be used with the Lucid M1 System and cannot operate independently. The raTCD is a modification of a 510k cleared device called NeuralBot Guided Headmount Accessory (K180455;22May2018). The NeuralBot Investigational System is the investigational study device used in this study.

The NeuralBot Investigational System moves two ultrasound probes around the two temporal regions (Right and Left) of the head to find the transtemporal window and then optimizes cerebral blood flow velocity measurements. The system uses TCD data to systematically specify and evaluate probe positions. The NeuralBot Investigational System consists of a head-support structure that houses two probe positioning modules, a robotic controller unit and computer tablets. The NeuralBot Investigational System is non-invasive and does not deliver energy into a subject.

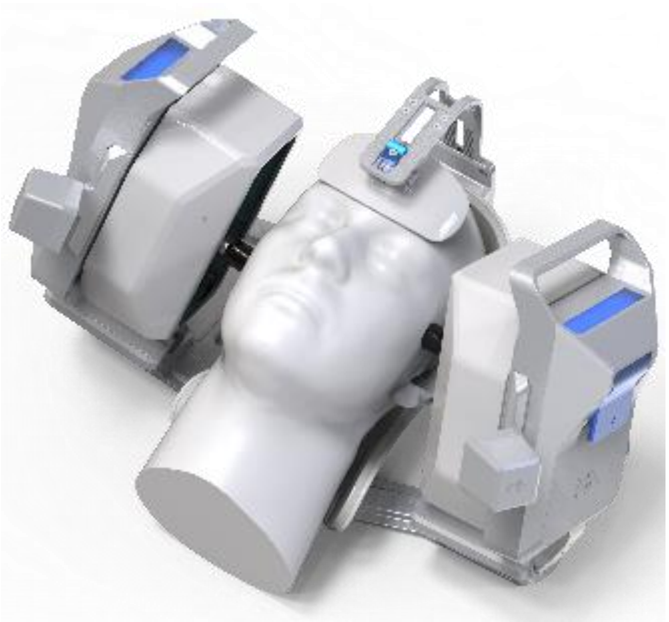

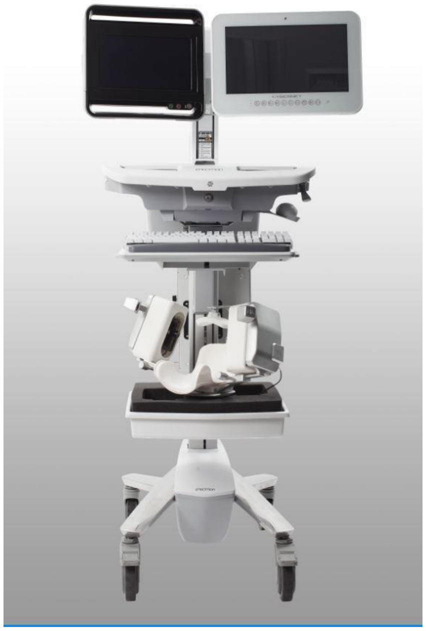

Supplemental Video: TTE negative, raTCD positive for large RLS

This video depicts a transthoracic echocardiogram (TTE, left) and robot-assisted transcranial Doppler (raTCD, right) in one of the study subjects with a negative TTE and a strongly positive raTCD. The four-chamber apical view of the TTE demonstrates opacification of the right atrium and ventricle (on the left of the figure) with agitated saline contrast and none of those bubbles crossing into the left atrium or ventricle, suggesting the absence of a RLS. In stark contrast, one can see and hear on the right part of the figure that many bubbles, generating characteristic “chirps” the white “streaks” seen on the raTCD – enough to fill entire cardiac cycles – indicating a large RLS. In the setting of discrepancy, the positive raTCD study is to be “trusted” as TTE is subject to false negative and the pattern of the result of raTCD does not suggest false positive. This is the audio/video version of Figure 2 within the manuscript.

Tables

Table 1 Subjects Enrolled by Study Site

	Enrolled (N=154)	Intent to Scan (N=129)	Per Protocol (N = 121)
Study Site, n (%)			
Barrow Neurological Institute	41 (27%)	35 (27%)	35 (29%)
CHI Memorial	70 (45%)	67 (52%)	61 (51%)
Houston Methodist	20 (13%)	13 (10%)	11 (9%)
Providence (two study locations, combined)	11 (7%)	4 (3%)	4 (3%)
Swedish Hospital	12 (8%)	10 (8%)	10 (8%)

Table 2.1 Imaging Accountability

	Intent to Scan (N=129)
Site Assessment by Imaging Type, n (%)	
SOC TCD	21 (16.3%)
SOC TTE	129 (100.0%)
SOC TEE	14 (11.1%)
Core Lab Assessment by Imaging Type, n (%)	
SOC TCD	18 (14.3%)
SOC TTE	129 (100.0%)
SOC TEE	14 (10.9%)

Legend: SOC = standard of care, TTE = transthoracic echocardiogram, TCD = transcranial Doppler, TEE = transesophageal echocardiogram

Table 2.2: Outcomes in subjects with TEE

Case	raTCD	raTCD	TTE	TTE	TCD	TCD	TEE	TEE
	RLS +/-	SLS Grade	RLS +/-	Bubbles	RLS +/-	SLS Grade	RLS +/-	Bubbles
**1**	+	1	-	N/A	-	0	-	N/A
**2**	+	1	+	>20	-	0	-	N/A
**3**	+	1	-	N/A	-	0	+	<10
**4**	+	1	-	N/A	+	1	-	N/A
**5**	+	5	+	10-20	+	5	+	10-20
**6**	-	0	-	N/A	-	0	-	N/A
**7**	+	1	-	N/A	N/A	N/A	-	N/A
**8**	+	5	+	>20	N/A	N/A	-	N/A
**9**	+	5	+	10-20	N/A	N/A	+	<10
**10**	+	4	+	<10	N/A	N/A	+	<10
**11**	+	4	-	N/A	N/A	N/A	+	<10
**12**	+	1	-	N/A	N/A	N/A	-	N/A
**13**	+	2	-	N/A	N/A	N/A	+	<10
**14**	-	0	-	N/A	N/A	N/A	-	N/A

Legend: raTCD = robot-assisted transcranial Doppler, TTE = transthoracic echocardiogram, TCD = transcranial Doppler, TEE = transesophageal echocardiogram, RLS = right to left shunt, SLS = Spencer Logarithmic Scale, N/A = not applicable

Table 2.3 RLS detection accuracy: raTCD vs TEE

raTCD vs TEE			
	TTE+	TTE-	**Total**
raTCD+	6	6	**12**
raTCD-	0	2	**2**
**Total**	**6**	**8**	

Legend: raTCD = robot-assisted transcranial Doppler, TEE = transesophageal echocardiogram

Table 2.4 RLS detection accuracy: TTE vs TEE

TTE vs TEE			
	TEE+	TEE-	**Total**
TTE+	3	2	**5**
TTE-	3	6	**9**
**Total**	**6**	**8**	

Legend: raTCD = robot-assisted transcranial Doppler, TTE = transthoracic echocardiogram, TEE = transesophageal echocardiogram

Table 2.5 RLS detection accuracy: raTCD vs TTE

	raTCD	TTE
Sensitivity (%)	100	50
Specificity (%)	25	75
PPV (%)	50	60
NPV (%)	100	67

Legend: raTCD = robot-assisted transcranial Doppler, TTE = transthoracic echocardiogram, TEE = transesophageal echocardiogram, PPV = positive predictive value, NPV = negative predictive value

Table 2.6 RLS % detection: raTCD vs TEE

	**TEE (Site Assessment)**			**TEE (Core Lab)**		
**raTCD**	**Positive**	**Negative**	**Total**	**Positive**	**Negative**	**Total**
**Positive**	7 (50.0%)	5 (35.7%)	12 (85.7%)	6 (42.9%)	6 (42.9%)	12 (85.7%)
**Negative**	1 (7.1%)	1 (7.1%)	2 (14.3%)	0 (0%)	2 (34.9%)	2 (14.3%)
**Total**	8 (57.1%)	6 (42.9%)		6 (42.9%)	8 (57.1%)	
**Difference**	28.6%	95% CI 11.7%-54.6%	p = 0.221	42.9%	95% CI 21.4%-67.4%	p = 0.041

Legend: raTCD = robot-assisted transcranial Doppler, TEE = transesophageal echocardiogram

Table 2.7 RLS % detection: raTCD vs TCD

	**TCD (Site Assessment)**		
**raTCD**	**Positive**	**Negative**	**Total**
**Positive**	12 (57.1%)	6 (28.6%)	18 (85.7%)
**Negative**	0 (0.0%)	3 (14.3%)	3 (14.3%)
**Total**	12 (57.1%)	9 (42.9%)	
**Difference**	28.6%	95% CI 13.8%-50.0%	p = 0.041

Legend: raTCD = robot-assisted transcranial Doppler, TCD = transcranial Doppler
N.B.: There is no TCD Core Lab assessment due to the limitation of the variability in reporting standards of various TCD instruments utilized by the participating clinical sites. Not all instruments were able to deliver reported data in a way that allowed for structured review according to study protocol.

Table 3: Transtemporal window(s)

	ITS	PP
Both present	106	100
Unilateral left present	3	3
Unilateral right present	11	11
None present	9	7

Table 4: Success rate of raTCD

	ITS	PP
Failed registrations	1	0
Bilateral signal absence	9	9
Unilateral signal absence	17	16
Complete study	129	121
Device malfunction	5	5

RoBotic TCD Ultrasound BubbLe Study Compared to Transthoracic Echocardiography for Detection of Right to Left Shunt

The BUBL Study

Date: 02 AUG 2021

Version: 05

Protocol No.: NA-07BBL-01

Sponsor: NovaSignal Corp.
2440 S. Sepulveda Blvd., Suite 115
Los Angeles, CA 90064

Revision History:

Version Date	Description
Version 01 – 21MAY2020	Original Document – not implemented
Version 02 – 15JUN2020	Amendment to add exclusion criteria #2 for pregnant women per WIRB recommendation
Version 03 – 11AUG2020	Administrative change from Neural Analytics to NovaSignal company name change
Version 04 – 12FEB2021	Removed upper limit of age Inclusion Criteria. Added continuation of enrollment after the first 150 subjects with TTE are enrolled up to another 150 subjects with TEE performed
Version 05 – 02AUG2021	Revised Sub-Study to include TTE or TEE after Main Study 150 subjects are enrolled. Removed single-site sub-study at UTHSC.

Confidentiality Statement

The information contained in this document, especially unpublished data, is confidential and the proprietary property of NovaSignal. It is therefore provided to you in confidence as an investigator, potential investigator, or consultant, for review by you, your staff, and if necessary, an Institutional Review Board. It is understood that this information will not be disclosed to others without written authorization from NovaSignal except to the extent necessary to obtain informed consent from those persons who may receive the medical device.

Protocol Signature Page- Principal Investigator

I have read the protocol entitled, *Robotic TCD Ultrasound Bubble Study Compared to Transthoracic Echocardiography for Detection of Right to Left Shunt,* and agree that it contains all necessary details for carrying out the described study. I will conduct this study as outlined therein and will make reasonable effort to complete the study within the designed time frame. I will provide copies of the study plan and all information furnished by NovaSignal to all study personnel under my supervision. I will discuss this material with them to assure that they are fully informed about the device and the conduct of this study.

I will ensure that the study is conducted according to applicable regulations, to applicable laws and to hospital policy and Institutional Review Board (IRB) and/or Ethics Committee (EC) requirements.

______________________	______________________	_______/________/________
Principal Investigator's Printed Name	Principal Investigator's Signature	Date (dd/mmm/yyyy)

Protocol Signature Page- Sub-Investigator

I have read the Study entitled, *Robotic TCD Ultrasound Bubble Study Compared to Transthoracic Echocardiography for Detection of Right to Left Shunt,* and agree that it contains all necessary details for carrying out the described study.

I will ensure that the study is conducted according to applicable regulations, to applicable laws and to hospital policy and Institutional Review Board (IRB) and/or Ethics Committee (EC) requirements.

______________________	______________________	_______/_______/_______
Sub- Investigator's Printed Name	Sub-Investigator's Signature	Date (dd/mmm/yyyy)

TABLE OF CONTENTS

1 PROTOCOL SYNOPSIS 7
1.1 LIST OF ABBREVIATIONS 13
2 BACKGROUND AND RATIONALE OF STUDY 14
2.1 BACKGROUND INFORMATION 14
2.2 CURRENT OPTIONS 14
2.3 LIMITATIONS 14
2.4 RATIONALE 15
2.5 POTENTIAL RISKS AND BENEFITS 15
3 DEVICE DESCRIPTION 15
3.1 LUCID M1 TRANSCRANIAL DOPPLER SYSTEM 15
3.2 NEURALBOT INVESTIGATIONAL SYSTEM (NB-IS) 16
3.3 INTENDED USE OF THE DEVICE 16
3.4 CONTRAINDICATIONS 17
3.5 STUDY DEVICE TRAINING REQUIREMENTS 17
3.6 DEVICE STORAGE AND ACCOUNTABILITY 18
4 STUDY OBJECTIVES 18
4.1 PRIMARY EFFICACY ENDPOINT 18
4.2 SECONDARY TECHNICAL EFFICACY ENDPOINTS: 18
4.3 EXPLORATORY ENDPOINTS: 18
4.4 PRIMARY SAFETY ENDPOINT: 18
4.5 SECONDARY SAFETY ENDPOINTS: 18
5 STUDY DESIGN 19
5.1 STUDY POPULATION SIZE 19
5.2 STUDY DURATION 19
5.3 STUDY ENTRY CRITERIA – TTE MAIN STUDY 19
5.3.1 Inclusion Criteria 19
5.3.2 Exclusion Criteria 19
5.4 STUDY ENTRY CRITERIA – TTE OR TEE SUB-STUDY (AFTER FIRST 150 SUBJECTS WITH TTE ENROLLED) 20
5.4.1 Inclusion Criteria 20
5.4.2 Exclusion Criteria 20
6 STUDY PROCEDURES 20
6.1 INFORMED CONSENT AND SCREENING 20
6.2 NUMBERING OF STUDY SUBJECTS 21
6.3 SCREEN FAILURES 21
6.4 STUDY DISCONTINUATION BY IRB OR SPONSOR 21
6.5 STUDY SCHEDULE 22
6.5.1 Schedule of Assessments 22
6.6 SCANNING/DATA ACQUISITION 22
6.7 NEURALBOT INVESTIGATIONAL SYSTEM (NB-IS) SCANNING TECHNIQUE 22
6.8 ALARA CONSIDERATIONS 23
6.9 STANDARD OF CARE PROCEDURES 24
6.10 STUDY-SPECIFIC PROCEDURES 24
6.11 SCREENING PROCEDURES 24
6.12 PROCEDURE –NB-IS TCD SCANNING SESSION 24

6.12.1 Patient Preparation – Valsalva Maneuver (VM) ... 24
6.12.2 Contrast Agent Preparation ... 24
6.12.3 SOC Transcranial Doppler Ultrasound Procedure (TCD) (as applicable) ... 25
6.12.4 NeuralBot Investigational System (NB-IS) TCD Procedure ... 25
6.12.5 Transthoracic Echocardiography Procedure (TTE) per SOC ... 26
6.12.6 Transesophageal Echocardiography Procedure (TEE) per SOC (as applicable) ... 26
6.12.7 Follow-Up Procedures ... 27
6.13 NB-IS TCD DATA COLLECTION AND DATA TRANSFER ... 27
6.14 STUDY WORKFLOW ... 28

7 RISKS AND BENEFITS ... 29

7.1 KNOWN POTENTIAL RISKS ... 29
7.2 BENEFITS ... 29

8 SAFETY ASSESSMENTS ... 29

8.1 SAFETY PARAMETERS ... 29
8.1.1 Adverse Event (AE) ... 29
8.1.2 Serious Adverse Event (SAE) ... 30
8.1.3 Unanticipated Adverse Device Effect (UADE/USADE) ... 30
8.1.4 Device Deficiency, Device Malfunction, and Use Error ... 30
8.2 REPORTING DEVICE AND/OR PROCEDURE RELATED ADVERSE EVENTS ... 31
8.3 CLASSIFICATION OF A DEVICE AND/OR PROCEDURE RELATED ADVERSE EVENT ... 32
8.3.1 Severity Rating of Event ... 32
8.3.2 Relationship of Study Device and/or Procedure ... 32
8.3.3 Outcome of Event ... 32
8.4 REPORTING PROCEDURES ... 33

9 PROTOCOL DEVIATIONS ... 33

10 QUALITY CONTROL AND QUALITY ASSURANCE ... 34

10.1 ORGANIZATIONAL PREPARATIONS ... 34
10.2 TRAINING ... 34
10.3 DATA QUALITY ASSURANCE ... 34
10.4 SUBJECT PRIVACY ... 34
10.5 CASE REPORT FORMS ... 35
10.6 INVESTIGATIONAL DEVICE ACCOUNTABILITY ... 35
10.7 SELECTION OF INVESTIGATORS ... 35
10.8 CLOSE-OUT DOCUMENT REVIEW ... 35

11 STATISTICAL ANALYSIS ... 35

11.1 PRIMARY ENDPOINT ANALYSIS ... 35
11.2 SECONDARY TECHNICAL EFFICACY ENDPOINT(S) ANALYSIS ... 36
11.3 EXPLORATORY ENDPOINTS ANALYSIS ... 36
11.4 IMAGING CORE LAB ANALYSIS ... 37
11.5 RLS/PFO VALIDATION AND ANALYSIS ... 37
11.6 SAMPLE SIZE CALCULATION ... 37
11.7 PRIMARY SAFETY ENDPOINT ANALYSIS ... 39
11.8 SECONDARY SAFETY ENDPOINT ANALYSIS ... 39

12 ETHICAL CONSIDERATIONS ... 39

12.1 INVESTIGATIONAL REVIEW BOARD (IRB) APPROVAL ... 39

12.2 Role of NovaSignal 39
12.3 Investigator Responsibilities 40
12.4 Records Custody 40

13 APPENDIX 41

13.1 Spencer Logarithmic Scale (SLS)[10] 41
13.2 International Consensus Criteria (ICC)[11] 41
13.3 Classification of Potential Causative Mechanism in PFO–Associated Stroke[1] 41

14 REFERENCES 42

1 PROTOCOL SYNOPSIS

**Protocol Synopsis:**	
**Study Sponsor:**<br>NovaSignal Corp.<br>2440 S. Sepulveda Blvd., Ste. 115<br>Los Angeles, CA 90064	**Protocol No.:** NA-07BBL-01<br>**Regulatory Class:** II<br>**Study Device(s):** *NeuralBot Investigational System (NB-IS)*<br>**Development Phase:** Pivotal
**Study Title:**<br>Robotic TCD Ultrasound Bubble Study Compared to Transthoracic Echocardiography for Detection of Right to Left Shunt	
**Overall Study Design:**<br>This study is a multi-center, prospective, single-arm, non-significant risk (NSR) device study in which up to 150 evaluable subjects with embolic stroke of undetermined source (ESUS) according to standardized criteria[13] will be evaluated with NB-IS TCD and standard of care TTE to screen for right to left shunt (RLS) or patent foramen ovale (PFO). Additionally, up to 150 evaluable subjects will be evaluated with NB-IS TCD and standard of care TTE or TEE.	
**Objective:**<br>To evaluate the shunt detection rate of the NeuralBot Investigational System (NB-IS) TCD relative to standard of care diagnostic techniques (transthoracic echocardiography (TTE), transesophageal echocardiography (TEE), and standard transcranial Doppler ultrasound (TCD) and to assess the safety, accuracy and usability of the NB-IS device.	
**Number of Subjects:**<br>TTE Main Study: Up to 150 subjects with suspected RLS/PFO receiving TTE<br>TEE Sub-Study: Up to 150 subjects with suspected RLS/PFO receiving TTE or TEE	**Number of Sites:**<br>Up to 7 centers total (US)
**Duration of Study Participation:**<br>• Enrollment: approximately 9-15 months<br>• Participant duration: 1-3 days (screening, NB-IS TCD, TCD, TTE, TEE); 1-60 days (follow-up)	
**<u>Primary Efficacy Endpoint:</u>**<br>• % detection of RLS/PFO with NB-IS TCD compared against standard of care Transthoracic Echocardiography (TTE)<br><br>**<u>Secondary Technical Efficacy Endpoint:</u>**<br>• % agreement for detection of RLS/PFO with NB-IS TCD vs SOC TCD<br>• % agreement for detection of RLS/PFO with NB-IS TCD vs Transesophageal Echocardiography (TEE)<br>• % agreement for detection of intervenable shunts with NB-IS TCD vs TTE<br>• No window rate (including both unilateral and bilateral absent acoustic windows)<br>• Success rate of the NB-IS TCD	

**Protocol Synopsis:**	
**Study Sponsor:**<br>NovaSignal Corp.<br>2440 S. Sepulveda Blvd., Ste. 115<br>Los Angeles, CA 90064	**Protocol No.:** NA-07BBL-01<br>**Regulatory Class:** II<br>**Study Device(s):** *NeuralBot Investigational System (NB-IS)*<br>**Development Phase:** Pivotal

- Incidence of device malfunctions

Exploratory Endpoints:

- Development of an automated algorithm for Spencer Scale and ICC grading
- Single site sub-study (UTHSC) of right atrial monitoring for timing of Valsalva during NB-IS and standard TCD, comparing shunt detection rate with/without right atrial (RA) monitoring against one another and each against TEE.

Primary Safety Endpoint:

- Incidence of device-related serious adverse events.

Entry Criteria TTE Main Study:

Inclusion Criteria

1. Subject 18 years of age and older.
2. Subject presents with a clinical condition characterized by neurological signs and symptoms that, in the opinion of the investigator, include embolic stroke or TIA in the differential diagnosis.
3. Scheduled for a transthoracic echocardiograph (TTE) study with agitated saline contrast (bubble study) per standard of care within ±30 days of informed consent.
4. Subject is able to successfully perform a Valsalva Maneuver (VM).
5. Subject or Legally Authorized Representative has the ability to provide informed consent and comply with the protocol.

Exclusion Criteria

1. Subject has undergone a right to left shunt (RLS) or patent foramen ovale (PFO) closure.
2. Female who is pregnant or lactating at time of admission
3. Subjects who underwent partial or full craniotomy/craniectomy within the past 6 months.
4. Subjects who have a physical limitation preventing TCD headset placement

Entry Criteria TTE or TEE Sub-Study (after first 150 subjects with TTE enrolled):

Inclusion Criteria

1. Subject 18 years of age and older.
2. Subject presents with a clinical condition characterized by neurological signs and symptoms that, in the opinion of the investigator, include embolic stroke or TIA in the differential diagnosis.
3. Scheduled for a transthoracic echocardiograph (TTE) or transesophageal echocardiograph (TEE) study with agitated saline contrast (bubble study) per standard of care within ±30 days of informed consent.
4. Subject is able to successfully perform a Valsalva Maneuver (VM).

**Protocol Synopsis:**	
**Study Sponsor:**<br>NovaSignal Corp.<br>2440 S. Sepulveda Blvd., Ste. 115<br>Los Angeles, CA 90064	**Protocol No.:** NA-07BBL-01<br>**Regulatory Class:** II<br>**Study Device(s):** *NeuralBot Investigational System (NB-IS)*<br>**Development Phase:** Pivotal

5. Subject or Legally Authorized Representative has the ability to provide informed consent and comply with the protocol.

Exclusion Criteria

1. Subject has undergone a right to left shunt (RLS) or patent foramen ovale (PFO) closure.
2. Female who is pregnant or lactating at time of admission
3. Subjects who underwent partial or full craniotomy/craniectomy within the past 6 months.
4. Subjects who have a physical limitation preventing TCD headset placement

Data Collection:

- Informed Consent
- Subject Demographics
- Relevant Medical History
- Physical Exam (SOC)
- NB-IS TCD Operator (name)
- NB-IS TCD Scan information
  - Date/Time of NB-IS TCD bubble study
  - NB-IS TCD scan Start/Stop times with and without VM
  - Saline/Air Injection(s) Start/Stop time(s) with and without VM
  - Head inclination (degree)/subject positioning/location of the exam
- SOC Transcranial Doppler Ultrasound (TCD) local results (SOC), if performed
  - Date/Time of SOC TCD bubble study
  - SOC TCD scan Start/Stop times with and without VM (as applicable)
  - Saline/Air Injection(s) Start/Stop time(s) with and without VM (as applicable)
  - Head inclination (degree)/subject positioning/location of the exam
- Transthoracic Echocardiography (TTE) local results (SOC)
  - Date/Time of TTE bubble study
  - TTE Start/Stop times with and without VM (as applicable)
  - Saline/Air Injection(s) Start/Stop time(s) with and without VM (as applicable)
- Transoesophageal Echocardiography (TEE) local results (SOC), if performed
  - Date/Time of TEE exam
  - TEE Start/Stop times (as applicable)
  - Saline/Air Injection(s) Start/Stop time(s) with and without VM (as applicable)
- Local Spencer Logarithmic Scale (SLS) grades (SOC TCD only)
- Local International Consensus Criteria (ICC) grades (SOC TCD only)
- Classification of Potential Causative Mechanism in PFO–Associated Stroke[1]
- PFO Closure Decision (post diagnostic procedures)
- PFO Closure Procedure, if performed

**Protocol Synopsis:**	
**Study Sponsor:**<br>NovaSignal Corp.<br>2440 S. Sepulveda Blvd., Ste. 115<br>Los Angeles, CA 90064	**Protocol No.:** NA-07BBL-01<br>**Regulatory Class:** II<br>**Study Device(s):** *NeuralBot Investigational System (NB-IS)*<br>**Development Phase:** Pivotal

- Relevant Concomitant Medications
- Device and/or Procedure Related Adverse Events (NB-IS and/or NB-IS TCD procedure)
- Device Deficiencies / Malfunctions / Technical Observations

Imaging Core Lab Analysis:

All imaging data will be sent to a core laboratory which will provide independent quantitative and qualitative assessment of all NB-IS TCD and TTE, and SOC TCD and TEE bubble study data, if performed. They will be blinded to the study and local diagnostic report data and provide independent review.

Data Analysis:

Evaluation of correlation between diagnostic imaging standard of care (local TCD/TTE/TEE) and NB-IS TCD diagnostic performance by an independent core laboratory. Success criteria will include clinical validation of NB-IS TCD for RLS/PFO detection targeting (i) 40% increase in NB-IS TCD sensitivity compared to TTE sensitivity and (ii) NB-IS TCD sensitivity ≥ 90% compared to SOC TCD sensitivity.

Schedule of Assessments			
**Assessments**	**Screening[1]**	**Procedure[1]**	**Follow-Up[1]**
Informed Consent	**X**		
Demographics	**X**		
Relevant Medical History	**X**		
Physical Exam ♦	**X**		
SOC TCD Bubble Study Exam ♦		**O[4]**	
NB-IS TCD Bubble Study Exam		**X[2]**	
Transthoracic Echocardiograph (TTE) Bubble Study ♦		**X[3]**	
Transesophageal Echocardiograph (TEE) ♦		**O[4] / X[8]**	
PFO Closure Decision ♦			**X[5]**
Classification of Potential Causative Mechanism in PFO–Associated Stroke[7]♦			**X**
PFO Closure Procedure ♦			**O[6]**
Device-Related Adverse Events		**X**	
Procedure-Related Adverse Events		**X**	
Concomitant Medications	**X**	**X**	
Device Deficiencies		**X**	

♦ Standard of Care **X** = Required **O** = Optional, if performed

1. Screening, Procedure, and Follow-Up Visits can occur on the same day
2. NB-IS TCD performed with contrast agent of 9 ml bacteriostatic saline with 1 ml air mixed 10 times. Performed at Rest AND with VM – 60 sec recordings each in supine positions AND with HOB raised to 45 degrees.
3. TTE can be performed within ±30 days of informed consent per SOC.
4. SOC TCD and TEE results collected if performed as applicable per standard of care.
5. PFO closure decision collected post diagnostic imaging procedures.
6. PFO closure procedure data collected within 60 days post diagnostic workup, if performed
7. Elgendy AY, Saver JL, Amin Z, et al; MSCAI14. Proposal for Updated Nomenclature and Classification of Potential Causative Mechanism in Patent Foramen Ovale-Associated Stroke. JAMA Neurol. 2020 Apr 13. doi:10.1001/jamaneurol.2020.0458. [Epub ahead of print] PubMed PMID: 32282016**.**
8. SOC TTE or TEE will be required after the first 150 TTE subjects are enrolled and can be performed within ±30 days of informed consent per SOC.

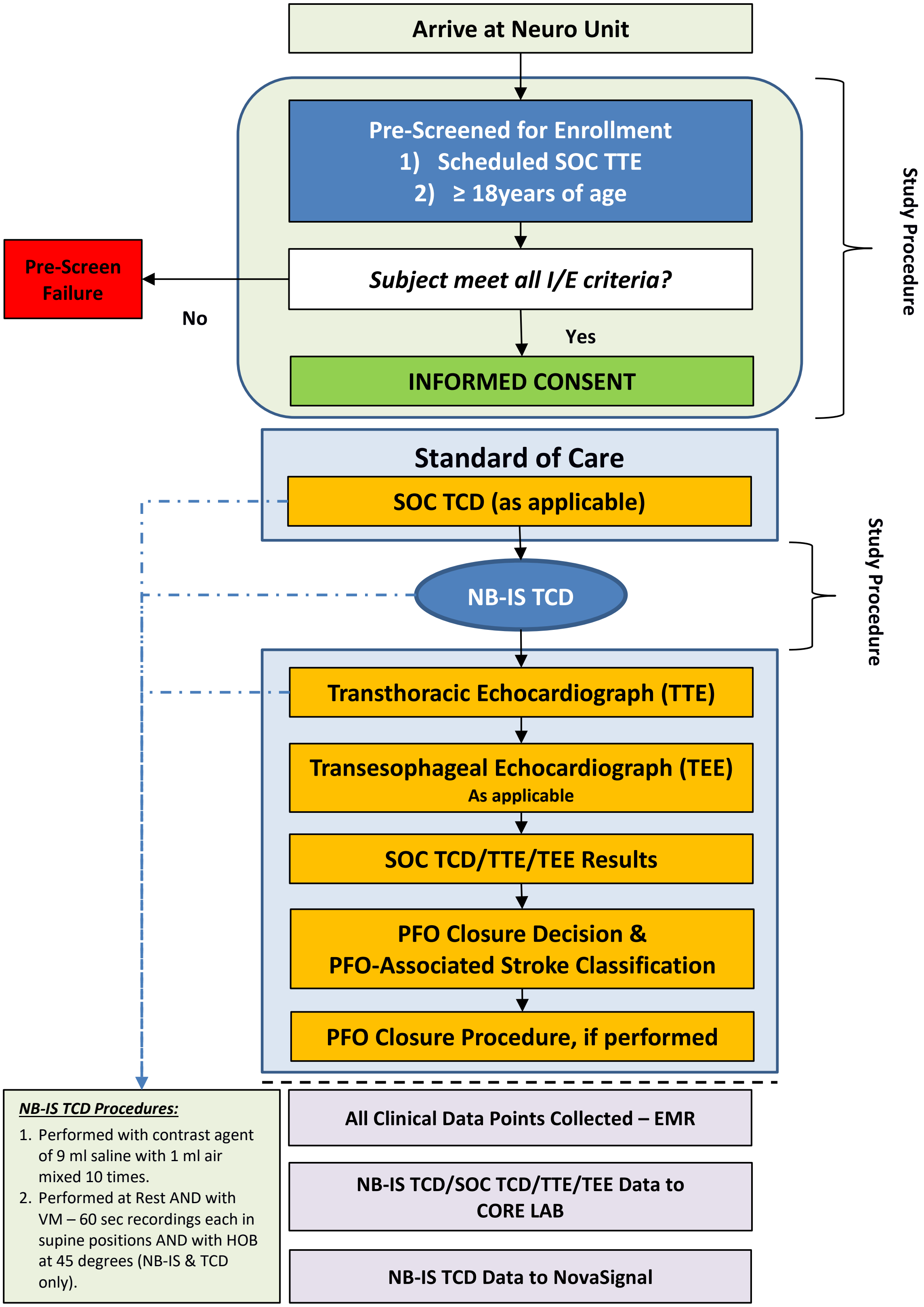
Arrive at Neuro Unit
Pre-Screened for Enrollment
1) Scheduled SOC TTE
2) ≥ 18years of age
Subject meet all I/E criteria?
Pre-Screen Failure
No
Yes
INFORMED CONSENT
Study Procedure
Standard of Care
SOC TCD (as applicable)
NB-IS TCD
Study Procedure
Transthoracic Echocardiograph (TTE)
Transesophageal Echocardiograph (TEE)
As applicable
SOC TCD/TTE/TEE Results
PFO Closure Decision &
PFO-Associated Stroke Classification
PFO Closure Procedure, if performed
NB-IS TCD Procedures:
1. Performed with contrast agent of 9 ml saline with 1 ml air mixed 10 times.
2. Performed at Rest AND with VM – 60 sec recordings each in supine positions AND with HOB at 45 degrees (NB-IS & TCD only).
All Clinical Data Points Collected – EMR
NB-IS TCD/SOC TCD/TTE/TEE Data to CORE LAB
NB-IS TCD Data to NovaSignal

1.1 List of Abbreviations

Abbreviation/Term	Definition
AE	Adverse Event
CBF	Cerebral Blood Flow
CBFV	Cerebral Blood Flow Velocity
CFR	Code of Federal Regulations
CE	Conformité Européenne
CT Scan	Computed Axial Tomography Scan
DM	Device Malfunction
EC	Ethics Committee
eCRF	Electronic Case Report Form
EDC	Electronic Data Capture
ESUS	Embolic Stroke of Undetermined Source
FDA	Food and Drug Administration
GCP	Good Clinical Practice
HIPAA	Health Insurance Portability and Accountability Act
HOB	Head of Bed
ICF	Informed Consent Form
IRB	Institutional Review Board
MCA	Middle Cerebral Artery
PFO	Patent Foramen Ovale
RLS	Right to Left Shunt
SAE	Serious Adverse Event
UADE	Unanticipated Adverse Device Effects
TCD	Transcranial Doppler
TEE	Transesophageal Echocardiograph
TTE	Transthoracic Echocardiograph
TIC	Thermal Index for Cranial Bone

2 BACKGROUND AND RATIONALE OF STUDY

2.1 Background Information

Right to Left shunt (RLS) through a Patent Foramen Ovale (PFO) is a known risk factor of ischemic stroke and is present in approximately 25% of the entire population. RLS is responsible for 43% of cryptogenic stroke in patients with a mean age of 54 years with no other risk factors[2].

The American College of Cardiology recognizes the benefit of procedures to close PFO in patients with PFO-associated stroke for secondary stroke prevention[3]. With the connection between PFO closure and prevention of secondary stroke, there is a need for assessment tools that are both non-invasive and accurate.

2.2 Current Options

In the assessment of a possible RLS, the European Society of Cardiology, the European Stroke Organization and the European Academy of Neurology recommend either transthoracic echocardiogram (TTE) or transcranial Doppler (TCD) imaging of patients with cryptogenic stroke or embolic stroke of undetermined source (ESUS)[4] that represents an updated classification of cryptogenic cerebral ischemia including a systematic and thorough diagnostic work-up. All of these diagnostic procedures involve the injection of agitated saline with the patient's performance of a Valsalva Maneuver (VM). While TTE and TCD are initial recommended imaging, the PFO presence and size should be subsequently assessed using the more invasive transesophageal echocardiography (TEE) methodology[5]. TTE and TEE are ultrasonic imaging methods used to evaluate the structure of the heart. TTE is performed non-invasively through the chest wall using a handheld transducer. TEE is slightly more invasive and performed under mild sedation, involving the guidance of an ultrasound probe down the esophagus for optimal imaging of the heart. Intra-cardiac echocardiography (ICE) is perhaps the most invasive imaging method and involves the insertion of a catheter to image the heart directly.

Outside of these structural imaging modalities, TCD involves the monitoring of blood flow in the brain; the presence of microbubbles on a TCD exam at rest and/or after VM could indicate the presence of RLS. In this way, TCD is able to mimic the occurrence of an ESUS in a diagnostic environment. TCD, like TTE, is non-invasive and involves probe placement and manipulation by an expert technician.

2.3 Limitations

Although TTE is the most frequently used non-invasive technique for the assessment of RLS, it is limited by its dependence on the expertise of the technician performing the exam. Furthermore, its moderate sensitivity (45.1%) but high specificity (99.6%) in RLS indication should not be relied upon in the determination of treatment for ESUS patients[6].

While TEE is considered the gold standard for visualization of the PFO after its presence has been indicated, its invasive nature can make it difficult for the patient to comply with the necessary steps for the VM. Although TEE is contraindicated in patients with dysphagia or esophageal issues, its sensitivity and specificity are 95% and 95% for indication of RLS[7].

Like TTE, the diagnostic value of TCD is limited by the expertise of the technician performing the exam and the presence of optimal acoustic windows. Signal acquisition is a well-documented obstacle in TCD evaluation regardless of pathology and is no different in the case of RLS

assessment[8]. However, unlike TTE, TCD has shown excellent sensitivity and specificity in RLS detection (96.1% and 92.4%, respectively) against the gold standard of TEE[6].

2.4 Rationale

The non-invasive nature and comparable performance to TEE make TCD a viable but under-utilized screening tool in the assessment of RLS. There exists a need to decrease its user-dependence in order to allow for its expanded application.

Despite TTE's superior specificity, the high sensitivity of TCD indicates its obvious diagnostic value in the detection of RLS[6].

2.5 Potential Risks and Benefits

TCD ultrasonography is a safe, non-invasive, and reproducible technique that allows the assessment of cerebral blood flow (CBF) in a person. TCD data collection relies on the level of experience of a TCD technologist to acquire an accurate and high-quality signal from the intracranial vessel(s) of interest, thus per user, the data quality is variable[9]. NovaSignal has developed the NeuralBot Investigational System (NB-IS) to assist in the TCD data collection such that consistent high-quality data may be obtained independent of the experience of the user.

NovaSignal will collect safety and technical feasibility data regarding the use of TCD ultrasound via the NeuralBot Investigational System (NB-IS) device in humans. Data collected during this study is for research purposes only. No treatment or healthcare decisions will be made based on study measurements collected in this study.

3 DEVICE DESCRIPTION

3.1 Lucid M1 Transcranial Doppler System

The Lucid M1 System is an adjunctive, portable, non-invasive, non-ionizing radiation, point-of-care TCD diagnostic ultrasound system. It is designed to non-invasively measure and display CBFV over the head and neck with a reusable, non-sterile 2-MHz hand-held probe. It can also be used bilaterally to monitor the blood flow velocity of the vessels insonated via the temporal window of the head with a headset with two reusable, non-sterile 2-MHz monitoring transducers. The system can also provide an emboli count for emboli detection.

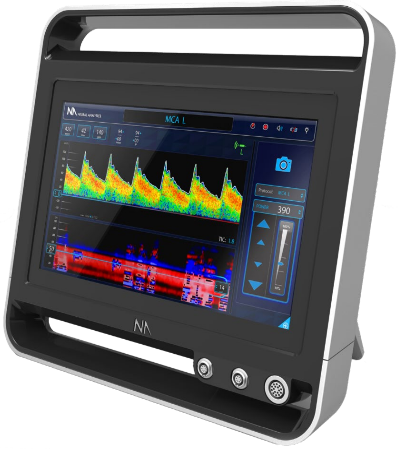

3.2 NeuralBot Investigational System (NB-IS)

The NeuralBot Investigational System (NB-IS) is comprised of both the Lucid M1 System and the NeuralBot accessory. The NeuralBot accessory must be used with the Lucid M1 System and cannot operate independently. The NB-IS is a modification of a 510k cleared device called NeuralBot Guided Headmount Accessory (K180455;22May2018). The NB-IS is the investigational study device used in this study.

The NB-IS moves two ultrasound probes around the two temporal regions (Right and Left) of the head to find the transtemporal window and then optimizes CBFV measurements. The system uses TCD data to systematically specify and evaluate probe positions. The NB-IS consists of a head-support structure that houses two probe positioning modules, a robotic controller unit and computer tablets. The NB-IS is non-invasive and does not deliver energy into a subject.

In this study, the NB-IS system will consist of study specific modifications to aid in study workflow. These modifications are not FDA cleared; however, the modifications do not impact the data collected in the study. This system will be referred to as NB-IS. The modifications do not introduce new risks or alter existing risks. All modifications will be tested to specifications per NovaSignal's Design Control standard operating procedures.

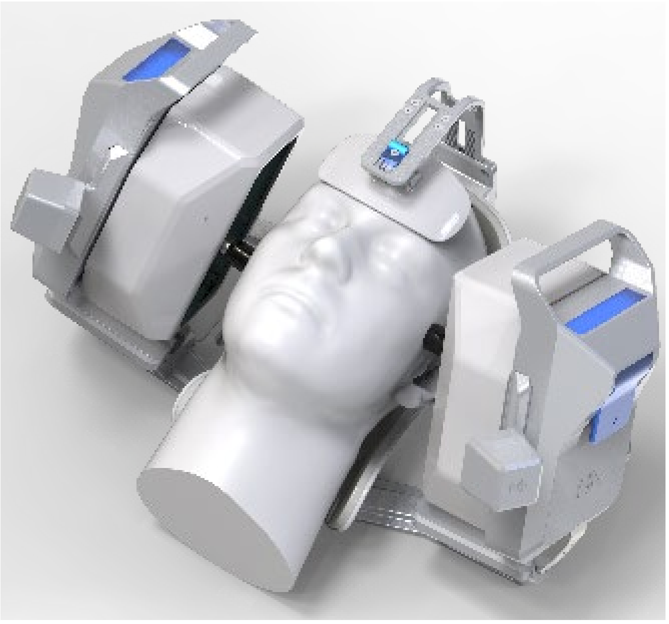

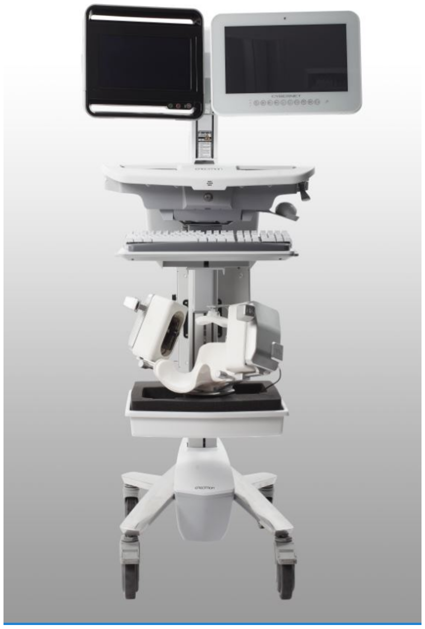

3.3 Intended use of the device

The NovaSignal Lucid M1 System is a medical ultrasound system intended for use as an adjunct to the standard clinical practices for measuring and displaying cerebral blood flow velocity within the major conducting arteries and veins of the head and neck. Additionally, the Lucid M1 System measures the occurrence of transient emboli signals within the blood stream.

The Lucid M1 System is an FDA cleared diagnostic medical device, Lucid M1 Transcranial Doppler Ultrasound System (510k K160442; CE Mark 32518: 21Oct2016).

The NeuralBot Investigational System when used with the Lucid M1 System is a medical ultrasound device which assists the user in the setup and acquisition of cerebral blood flow velocity via the patient's temporal windows. It is intended for use as an adjunct to standard clinical practices for measuring and displaying cerebral blood flow velocity and the occurrence of transient emboli within the blood stream.

The NeuralBot Investigational System is a modification of a 510k cleared device called NeuralBot Guided Headmount Accessory (K180455; 22May2018). The NeuralBot Investigational System is the investigational study device used in this study.

The NeuralBot Investigational System is intended to be used by persons qualified by training in its safe and effective use. The device is not intended to replace other means of evaluating vital patient physiological processes, is not intended to be used in fetal applications and is not intended to be used inside the sterile field.

The NeuralBot Investigational System is intended to be used on study subjects in the supine (0 degrees) or up to 45-degrees reclined position on a bed or gurney in a clinical setting. The device is NOT intended to be used simultaneously with head imaging equipment such as MRI and CT.

The NeuralBot Investigational System is for use in this study only and is not cleared for use by the FDA. It is labelled with the following statement:

CAUTION: Investigational device. Limited by Federal (or United States) law to investigational use only.

The use of the Lucid M1 System with the NeuralBot Investigational System in this study is not for the purposes of diagnosis. This device is being used solely for research to develop technology for assisted TCD signal acquisition. As such, the device is considered a Nonsignificant Risk Device under 21 CFR 812.3(m).

3.4 Contraindications

The Lucid M1 System is not intended to be used in fetal applications or inside a sterile field. Use proposed in this study is consistent with the current FDA cleared and CE Mark approved labeling.

The NeuralBot Investigational System is not intended to be used when the following conditions are present:

- The NeuralBot Investigational System is not intended to be used in persons younger than 18 years of age.
- The NeuralBot Investigational System is not intended to be used in fetal applications.
- The NeuralBot Investigational System is not intended to be used inside the sterile field.

3.5 Study Device Training Requirements

In addition to the protocol training, all planned NB-IS TCD operators will be required to undergo training via a device in-service, which includes detailed reviews of the Lucid M1 System and NB-IS User Manuals and the study device as well as hands-on practice scanning with volunteer subjects.

NovaSignal will provide training for the use of the NeuralBot Investigational System, in adherence to the Instructions for Use/User Manual. Documentation of training should be maintained by the Principal Investigator throughout the study. Principal investigators will also develop clinical guidance training modules so as to standardize the bubble study protocol, including ensuring and documenting adequate Valsalva.

3.6 Device Storage and Accountability

The NeuralBot Investigational System specific serial numbers must be documented at a study site by a designated person, handled and stored properly in a secured location in which only the study staff have access to. The Principal Investigator must maintain an accurate record of the status of the product(s) throughout the study. Investigators are responsible for appropriate logging of the devices used, verification of packing slip information (i.e. lot numbers and quantity shipped), date and identity that each device was used in the study, disposition information regarding disposal or return to the Sponsor.

4 STUDY OBJECTIVES

To evaluate the shunt detection rate of the NeuralBot Investigational System (NB-IS) TCD relative to standard of care diagnostic techniques (transthoracic echocardiography (TTE), transesophageal echocardiography (TEE), and manual transcranial Doppler ultrasound (TCD)) and to assess the safety, accuracy, and usability of the NB-IS device.

4.1 Primary Efficacy Endpoint

- % detection of RLS/PFO with NB-IS TCD compared against standard of care Transthoracic Echocardiography (TTE)

4.2 Secondary Technical Efficacy Endpoints:

- % agreement for detection of RLS/PFO with NB-IS TCD vs SOC TCD
- % agreement for detection of RLS/PFO with NB-IS TCD vs Transesophageal Echocardiography (TEE)
- % agreement for detection of clinically significant shunts with NB-IS TCD vs TTE
- No window rate (including both unilateral and bilateral absent acoustic windows)
- Success rate of the NB-IS TCD
- Incidence of NB-IS device malfunctions

4.3 Exploratory Endpoints:

- Development of an automated algorithm for Spencer Logarithmic Scale[10] and International Consensus Criteria grading of RLS[11]

4.4 Primary Safety Endpoint:

Incidence of device-related serious adverse events.

4.5 Secondary Safety Endpoints:

Incidence of procedure-related adverse events related to agitated saline injection before and after VM (headache, allergic reactions, new onset neurological deficit, ischemic stroke, TIA or

pulmonary embolism complicating agitated saline contrast injection, etc) will be closely monitored and prospectively collected[12].

5 STUDY DESIGN

This study is a multi-center, prospective, single-arm, non-significant risk (NSR) device study in which up to 150 evaluable subjects with embolic stroke of undetermined source (ESUS) according to standardized criteria[13] will be evaluated with NB-IS TCD and standard of care TTE to screen for right to left shunt (RLS) or patent foramen ovale (PFO). Additionally, up to 150 evaluable subjects will be evaluated with NB-IS TCD and standard of care TTE or TEE.

5.1 Study Population Size

To ensure the study is adequately powered, up to 150 subjects will be enrolled at up to 7 centers in the US. The estimation of this sample size has taken into account the prevalence of suboptimal transtemporal windows and potential dropouts.

Once the 150 TTE subjects are enrolled, the study will continue to enroll up to another 150 subjects that received a TTE or TEE until enough TTE subjects have been enrolled in the different grades of the International Consensus Criteria (0,1,2,3) across the TTE Main Study and TTE/TEE Sub Study combined.

5.2 Study Duration

The enrollment period will last up to 15 months. Subject's participation in the study will last from 1 to 60 days.

The study will be complete when all subjects have been enrolled and all data collected. The study can be terminated at any time, for any reason, by NovaSignal. Should this occur, the study investigator will be notified as soon as possible. The Principal Investigators will be responsible for informing their IRBs of the termination of the trial.

5.3 Study Entry Criteria – TTE Main Study

5.3.1 *Inclusion Criteria*

A subject must meet all of the following inclusions criteria to be enrolled in the study:

1. Subject 18 years of age and older.
2. Subject presents with a clinical condition characterized by neurological signs and symptoms that, in the opinion of the investigator, include embolic stroke or TIA in the differential diagnosis.
3. Scheduled for a transthoracic echocardiograph (TTE) study with agitated saline contrast (bubble study) within ±30 days of informed consent.
4. Subject is able to successfully perform a Valsalva Maneuver (VM).
5. Subject or Legally Authorized Representative has the ability to provide informed consent and comply with the protocol.

5.3.2 *Exclusion Criteria*

A subject cannot be enrolled in the study if any of the following exclusion criteria are met:

1. Subject has undergone an RLS/PFO closure.
2. Female who is pregnant or lactating at time of admission
3. Subjects who underwent partial or full craniotomy/craniectomy within the past 6 months.
4. Subjects who have a physical limitation preventing TCD/Headmount placement.

5.4 Study Entry Criteria – TTE or TEE Sub-Study (after first 150 subjects with TTE enrolled)

5.4.1 *Inclusion Criteria*

A subject must meet all of the following inclusions criteria to be enrolled in the study:

1. Subject 18 years of age and older.
2. Subject presents with a clinical condition characterized by neurological signs and symptoms that, in the opinion of the investigator, include embolic stroke or TIA in the differential diagnosis.
3. Scheduled for a transthoracic echocardiograph (TTE) or transesophageal echocardiograph (TEE) study with agitated saline contrast (bubble study) within ±30 days of informed consent.
4. Subject is able to successfully perform a Valsalva Maneuver (VM).
5. Subject or Legally Authorized Representative has the ability to provide informed consent and comply with the protocol.

5.4.2 *Exclusion Criteria*

A subject cannot be enrolled in the study if any of the following exclusion criteria are met:

1. Subject has undergone an RLS/PFO closure.
2. Female who is pregnant or lactating at time of admission
3. Subjects who underwent partial or full craniotomy/craniectomy within the past 6 months.
4. Subjects who have a physical limitation preventing TCD/Headmount placement.

6 STUDY PROCEDURES

6.1 Informed Consent and Screening

Subject identification and eligibility will be determined by the PI or study team based on their clinical assessment of the potential subject while that patient is undergoing standard of care for their condition.

Before participation in the study, candidates who may be eligible for the study will be provided with an informed consent according to 21 CFR 56 and 45 CFR 160-164 and guidelines of the Investigational Review Board of the institution at which the study is being conducted. Candidates will be given time to review the consent form and ask questions about the study. The investigator or delegated study staff is responsible for obtaining written informed consent from each potential study participant. Informed consent should be obtained, when required, in written format and using a form approved by the local IRB.

The subject must receive a copy of the signed and dated informed consent if requested. Waivers of consent will not be utilized in this study. The candidate or legal representative must sign the

consent form prior to enrollment. Candidates will also be required to sign a Health Insurance Portability and Accountability Act (HIPAA) form which includes a Privacy Rule. This rule gives special safeguards to Protected Health Information (PHI) that is identifiable or can be directly linked to the subject (e.g., social security number, name, birth date). This authorization may be part of the informed consent form or separate. If applicable to the investigator site location, candidates will also be required to sign the California Experimental Subject Bill of Rights.

A subject will be considered enrolled at the time of Informed Consent signature.

6.2 Numbering of Study Subjects

Each site will be assigned a site number at the beginning of the study and each enrolled subject will be assigned a subject number. The subject number will consist of the study number, the site number followed by a sequential number that begins with “001”.

6.3 Screen Failures

Subject eligibility will be determined by the PI or study team based on their clinical assessment of the potential subject while the patient is undergoing standard of care assessments for a stroke in the hospital.

For this protocol, a single measurement is defined as a collection of data at a single depth on a single side of the head. A scan is defined as a set of measurements which occur during a single data collection session and can include multiple measurements. A bilateral scan refers to measurements which are collected on each side of the head.

Subjects will be measured with NB-IS TCD and the de-identified data will be reviewed to determine the quality of the data for analysis. Subjects are considered evaluable if their NB-IS TCD data is of adequate quality and a complete data set is acquired. An adequate, evaluable, and complete study is defined for the purposes of this protocol as successful acquisition of a unilateral signal at 40-65mm depth range recorded for the duration of the bubble study in the Resting position and with Valsalva Maneuver. If the scan quality is adequate, the subject study data (via eCRF) and imaging will be collected and the NB-IS TCD utilized for analysis of the study endpoints. If the scan quality is inadequate, all subject clinical and imaging data will not be included in the final analysis.

6.4 Study Discontinuation by IRB or Sponsor

The IRB may choose to discontinue the study at any center(s) for which they granted approval if the:

- The research study is not conducted in accordance with the IRB requirements.
- The research study indicates unexpected serious harm to Subjects.

The Sponsor may choose to discontinue the study should the Sponsor discover additional information during the study that may cause harm to subject safety.

If the study is terminated prematurely or suspended, the Sponsor will promptly inform all clinical Investigators of the termination or suspension and the reason(s) for this. The IRB/EC will also be informed, either by the Sponsor or Investigator if a local IRB/EC is utilized, promptly and provided with the reasons(s) for the termination. If applicable, regulatory authorities will be informed.

6.5 Study Schedule

6.5.1 Schedule of Assessments

Schedule of Assessments			
**Assessments**	**Screening[1]**	**Procedure[1]**	**Follow-Up[1]**
Informed Consent	**X**		
Demographics	**X**		
Relevant Medical History	**X**		
Physical Exam ♦	**X**		
SOC TCD Bubble Study Exam ♦		**O[4]**	
NB-IS TCD Bubble Study Exam		**X[2]**	
Transthoracic Echocardiograph (TTE) Bubble Study ♦		**X[3]**	
Transesophageal Echocardiograph (TEE) ♦		**O[4] / X[8]**	
PFO Closure Decision ♦			**X[5]**
Classification of Potential Causative Mechanism in PFO–Associated Stroke[7]♦			**X**
PFO Closure Procedure ♦			**O[6]**
Device-Related Adverse Events		**X**	
Procedure-Related Adverse Events		**X**	
Concomitant Medications	**X**	**X**	
Device Deficiencies		**X**	

♦ Standard of Care **X** = Required **O** = Optional, if performed

1. Screening, Procedure, and Follow-Up Visits can occur on the same day
2. NB-IS TCD performed with contrast agent of 9 ml bacteriostatic saline with 1 ml air mixed 10 times. Performed at Rest AND with VM – 60 sec recordings each in supine positions AND with HOB raised to 45 degrees.
3. TTE can be performed within ±30 days of informed consent per SOC.
4. SOC TCD and TEE results collected if performed as applicable per standard of care.
5. PFO closure decision collected post diagnostic imaging procedures.
6. PFO closure procedure data collected within 60 days post diagnostic workup, if performed
7. Elgendy AY, Saver JL, Amin Z, et al; MSCAI14. Proposal for Updated Nomenclature and Classification of Potential Causative Mechanism in Patent Foramen Ovale-Associated Stroke. JAMA Neurol. 2020 Apr 13. doi:10.1001/jamaneurol.2020.0458. [Epub ahead of print] PubMed PMID: 32282016**.**
8. SOC TTE or TEE will be required after the first 150 TTE subjects are enrolled and can be performed within ±30 days of informed consent per SOC.

6.6 Scanning/Data Acquisition

The User Manual provided for the device details, in specific steps, the actions to be executed with the device for scanning/data acquisition. A general overview is provided below.

6.7 NeuralBot Investigational System (NB-IS) Scanning Technique

The NB-IS TCD scanning session will consist of an initial set-up and signal search (up to 20 minutes). During this time, the system will search for CBFV signals at depths between 40-65 mm. Once the signal is acquired, the system can monitor the signal for up to 1 hour. In this study, once

the MCA signal has been acquired unilaterally or bilaterally, subjects will be monitored for up to 20 minutes during the delivery of the contrast agent at Rest and with Valsalva Maneuver.

The head cradle of the NeuralBot Investigational System is positioned underneath the subject's head and secured to a fixed location (bed, chair, or gurney). The user will affix two registration dots on each side of subject's temples. The subject's head is then positioned into the head cradle. The TCD modules are aligned to the head such that the probes are in contact with the subject's temples (temporal window). The user will register the Headmount to the subject's specific head shape and size. The user will apply gel to improve signal quality. The NeuralBot Investigational System will collect data and indicate to the user the scan status.

Subjects will be evaluated during the scanning period per ALARA ("As Low As Reasonably Achievable") criteria. Scanning may not violate ALARA levels and will be immediately terminated if ALARA criteria reached during a scanning session.

6.8 ALARA Considerations

ALARA Considerations: "As Low As Reasonably Achievable – ALARA" principle for ultrasound to reduce the amount of total exposure to the subject without compromising exam quality.

Diagnostic ultrasound, including TCD, has been used clinically and in research for decades. In addition to the FDA guidance, there are several groups including the American Institute of Ultrasound in Medicine (AIUM), British Medical Ultrasound Society (BMUS), and the World Federation for Ultrasound in Medicine and Biology (WFUMB) that make recommendations on ultrasound safety for both fetal and non-fetal applications. These groups have focused on several areas of ultrasound safety including Thermal Index (TIC).

The FDA specifically references the AIUM for the ALARA recommendations for TIC and corresponding scan durations in the new guidance released in October 2017. Table 1 shows the recommended scan duration for a given TIC range. The ALARA recommendations are based on the TIC output by the device (which is displayed on the Lucid M1 System device at all times). TIC is defined as:

TIC – the thermal index for applications in which the ultrasound beam passes through bone near beam entrance into the body.

Thermal Index (TIC)	Recommended Duration (minutes)
>6.0	0
5.0-6.0	0.25
4.0-5.0	<1
3.0-4.0	<4
2.5-3.0	<15
2.0-2.5	<60
1.5-2.0	<120
<1.5	No Limit

Table 1 - Recommended maximum exposure duration and TIC ranges (except the eyes which are not evaluated in this protocol)

If a subject meets ALARA criteria at any time during a scanning session, the scan will be discontinued.

6.9 Standard of Care Procedures

Most procedures completed as part of this study are considered standard practice and include relevant medical and medication history, physical exam, SOC transcranial Doppler ultrasound (TCD) bubble study, transthoracic echocardiography (TTE) bubble study, transesophageal echocardiography (TEE) bubble study, and PFO closure (status), if performed.

This study is incorporating the use of NB-IS TCD bubble study in conjunction with standard of care procedures.

6.10 Study-Specific Procedures

The procedures that are study-specific and not part of standard of care for diagnosis and treatment of RLS and PFO include the use of the NB-IS TCD System bubble study.

6.11 Screening Procedures

The following data will be collected, and assessments performed after obtaining informed consent from eligible subjects and prior to the NB-IS TCD procedure.

- Informed Consent
- Subject Demographics
- Relevant Medical History
- Physical Exam (SOC)
- Relevant Concomitant Medications

6.12 Procedure –NB-IS TCD Scanning Session

The NB-IS TCD bubble study can be performed during the same session as SOC TCD bubble study is performed and preferably before TTE and TEE (if applicable) bubble studies are performed. If a TTE bubble study is performed before TCD bubble study, the SOC TCD operator will ensure he/she are blinded to the results of the TTE prior to performing TCD. The NB-IS TCD operator will perform scanning sessions as follows:

6.12.1 Patient Preparation – Valsalva Maneuver (VM)

The study subject will be trained in performance of the VM prior to conducting the SOC TCD (if performed) and NB-IS TCD bubble study in accordance with the training module developed by the principal investigators.

6.12.2 Contrast Agent Preparation

For TCD embolus detection, the contrast agent consisting of 9 mL bacteriostatic saline solution and 1 mL air should be mixed between two (2) 10-mL syringes connected by a 3-way stopcock at least ten (10) times and then vertically injected into (ideally) the right antecubital vein as a bolus both at Rest and mid-way through injection before a ten second Valsalva Maneuver (VM). The agitated saline should be mixed with 1 mL of blood prior to injection, if possible[14,15,16]. If Definity

(ultrasound contrast-agent containing perflutren lipid microspheres) is given during TTE or TEE prior to any TCD (NB-IS or SOC) *must* use different IV, wait up to 24h from Definity injection *and* record without injection (baseline recording) for 5 minutes prior to injecting air contrast (screen for Definity still circulating) before SOC TCD and NB-IS TCD bubble study sessions.

6.12.3 SOC Transcranial Doppler Ultrasound Procedure (TCD) (as applicable)

If SOC TCD bubble study is performed during subject participation in the study, preference is for this exam to be conducted in same session as the NB-IS TCD with the same individual administering the bubble injections with the SOC TCD bubble exam occurring before NB-IS TCD. The SOC TCD bubble study will be performed per standard of care, however, if possible, preference would be to collect measurements similar to NB-IS TCD exam (e.g., supine position at Rest and with VM, 45 degrees at Rest and with VM, etc.).

The following data points will be collected for the SOC TCD procedure:

- SOC transcranial Doppler Ultrasound (TCD) bubble study local results (SOC)
    - Date/time of SOC TCD bubble study
    - SOC TCD scan Start/Stop times with and without VM (as applicable)
    - Saline/Air Injection(s) Start/Stop time(s) with and without VM (as applicable)
    - Head inclination (degree)/subject positioning/location of the exam
    - Local Positive/Negative results for RLS/PFO
    - Local Spencer Logarithmic Scale (SLS) grade (with and without VM)
    - Local International Consensus Criteria (ICC) grade (with and without VM)
-

6.12.4 NeuralBot Investigational System (NB-IS) TCD Procedure

The NB-IS TCD bubble study session will be conducted beginning in the supine position with the same IV line.

The NB-IS TCD operator will setup the study subject in the headmount of the NeuralBot Investigational System as per Section 6.7 NeuralBot Investigational System Scanning Technique. When the signal search has completed and the optimal signal has been identified unilaterally or bilaterally , the NB-IS device will continue monitoring the signal(s) for up to 20 minutes during the delivery of the contrast agent at Rest and with VM.

First, 10 mL of mixed contrast agent will be injected into the right antecubital vein while the subject is at rest and the MCA signal recorded for a period of 60 seconds. The subject will remain at rest for approximately 3 to 5 minutes or when bubbles have cleared.

Second, another 10 mL of contrast agent will be injected in the right antecubital vein and the VM performed mid-way through injection, sustained for ten seconds. The MCA signal will be recorded for a period of 60 seconds; characteristic MCA waveform morphology change and mean velocity decrease of at least 25% will serve as proof of adequate Valsalva effort[17].

This procedure will be repeated with the study subject in the reclined position, with head of bed raised to 45 degrees where 10 mL contrast agent is injected when the subject is at rest and subsequently with a Valsalva Maneuver.

The following data points will be collected for the NB-IS TCD scanning session:

- Date/Time of NB-IS TCD bubble study
- NB-IS TCD Operator (name)
- NB-IS TCD Scan information
  - NB-IS TCD scan Start/Stop times with and without VM
  - Saline/Air Injection(s) Start/Stop time(s) with and without VM
  - Head inclination (degree)/subject positioning/location of the exam
- Relevant Concomitant Medications
- Device-Related Adverse Events (NB-IS)
- Procedure-related adverse events related to agitated saline injection before and after VM (headache, allergic reactions, new onset neurological deficit, ischemic stroke, TIA or pulmonary embolism complicating agitated saline contrast injection, etc) will be closely monitored and prospectively collected[18].
- Device Deficiencies / Malfunctions / Technical Observations

6.12.5 Transthoracic Echocardiography Procedure (TTE) per SOC

The TTE bubble study can be performed per standard of care within ±30 days of informed consent.

The following data points will be collected for the TTE procedure:

- Transthoracic Echocardiography (TTE) local results (SOC)
  - Date/Time of TTE bubble study
  - TTE Start/stop times with and without VM (as applicable)
  - Saline/Air Injection(s) Start/Stop time(s) with and without VM (as applicable)
  - Local Positive/Negative results for RLS/PFO
  - Local Bubble Count (as applicable)
  - Local TTE grading (as applicable)

6.12.6 Transesophageal Echocardiography Procedure (TEE) per SOC (as applicable)

The study subject, if referred per the institution's standard practice, will then undergo scheduled transesophageal echocardiography (TEE) bubble study with and without VM similarly to NB-IS TCD procedure.

The following data points will be collected for the TEE procedure, if performed:

- Transesophageal Echocardiography (TEE) local results (SOC) including shunt size and presence of atrial septal aneurysm.
  - Date/Time of TEE exam
  - TEE Start/Stop times with and without VM (as applicable)
  - Saline/Air Injection(s) Start/Stop time(s) with and without VM (as applicable)
  - Local Positive/Negative results for RLS/PFO
  - Local Bubble Count (as applicable)
  - Local TEE grading (as applicable)

6.12.7 Follow-Up Procedures

After the NB-IS TCD, TTE, SOC TCD and TEE (if performed) bubble study exams performed, the following data points will be collected:

- PFO Closure Decision (post-diagnostic procedure(s))
- Classification of Potential Causative Mechanism in PFO–Associated Stroke[1]

If a PFO closure procedure is performed within 60 days post diagnostic workup, the following data points will be collected:

- PFO Closure Procedure Data
- Shunt size
- Closure device(s) used

6.13 NB-IS TCD data collection and data transfer

NB-IS TCD data will be collected and stored on the NeuralBot Investigational System. The details of the data collection and transfer can be found in the device User Manual and Data Transfer procedures.

6.14 Study workflow

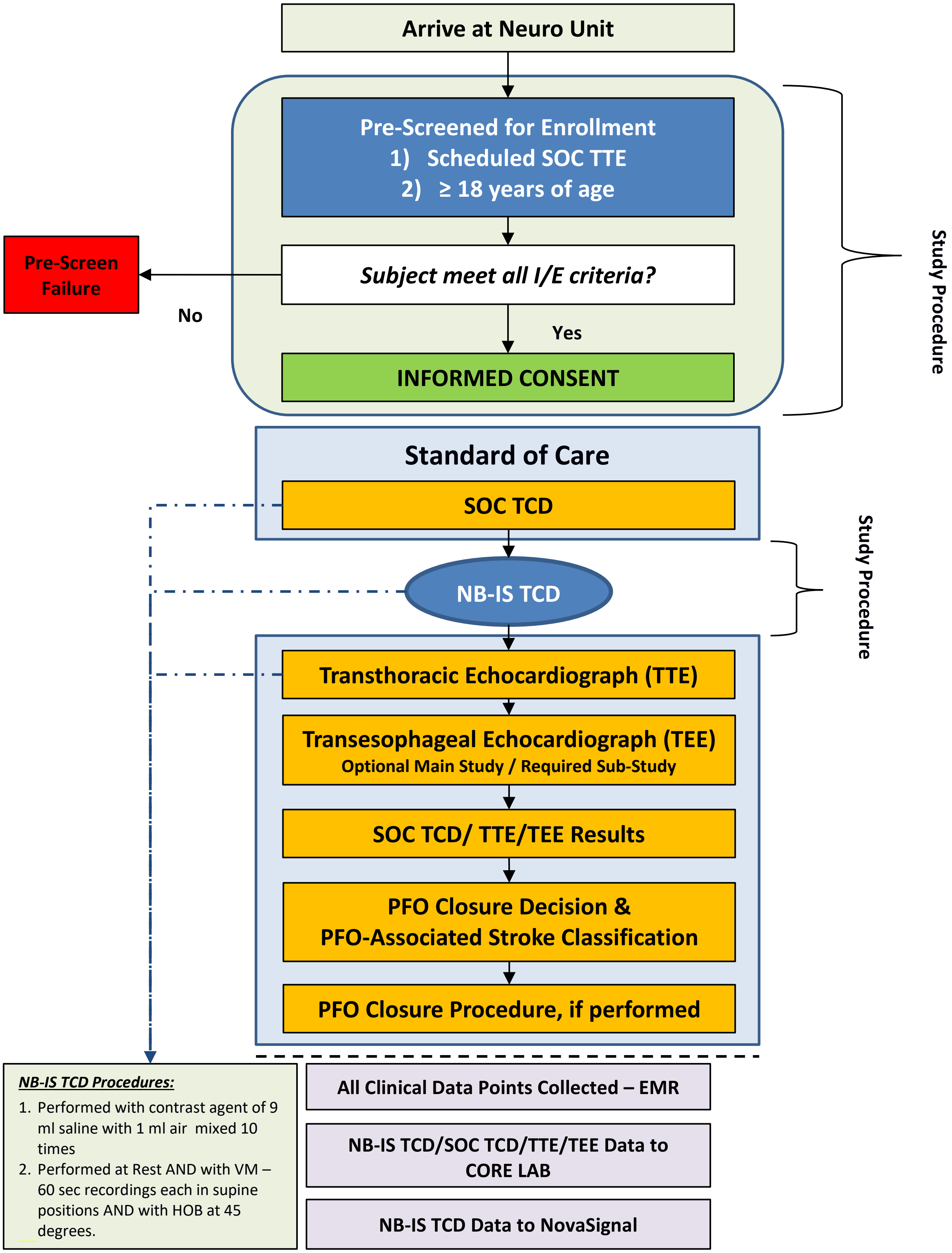

7 RISKS AND BENEFITS

There is minimal potential clinical risk associated with the scans described in the study. The risks associated with the use of the NeuralBot Investigational System in humans have not been determined as this study is one of the initial uses of the device. An ongoing study utilizing the device in subjects experiencing neurological symptoms of stroke has not reported any unexpected or serious device related adverse events (Protocol No.: NA-01STR-01). The NeuralBot Investigational System is designed to be physically and electro-mechanically safe according to manufacturing standards. At any time, for any reason, the use of the device can be discontinued.

7.1 Known Potential Risks

The following device-related adverse events have been identified as possible (anticipated) with the use of the NeuralBot Investigational System:

- Physical discomfort of the head and neck due to pressure from the probe, or probe accidentally coming in contact with ears, eyes or hair.
- Psychological feeling of claustrophobia- the fear of being enclosed in a small space.

7.2 Benefits

There is no direct benefit to the subject participating in the study.

8 SAFETY ASSESSMENTS

8.1 Safety Parameters

8.1.1 Adverse Event (AE)

An adverse event (AE) is defined as any untoward medical occurrence, unintended disease or injury, or untoward clinical signs (including abnormal laboratory findings) in subjects, users, or other person, whether or not related to the investigational medical device (ISO14155:2011E).

- Note 1: This definition includes events related to the investigational medical device or the comparator

- Note 2: This definition includes events related to the procedures involved

- Note 3: For users or other persons, this definition is restricted to events related to the study device.

For the purpose of this protocol, adverse events will be reported and recorded (via eCRF) if any of the following apply:

- Causality is related to:

    - **the study device**
    - **the study-related procedure (not standard of care)**
    - **if causality is unknown**

- The event is a serious adverse event (SAE) related to the study device or study-related procedure

Any medical condition that is present at the time informed consent is obtained or prior to the start of the study procedure will be considered as baseline and not reported as an AE. Such conditions should be added to the medical history, if not previously reported.

Collection of device and/or procedure-related adverse events will start after the time that informed consent form is obtained. Device and procedure-related adverse events will be monitored throughout the study.

8.1.2 Serious Adverse Event (SAE)

A serious adverse event (SAE) is defined (ISO14155:2011E) as an adverse event that:

a) Led to a death
b) Led to a serious deterioration in the health of the subject that:
    1) resulted in a life-threatening illness or injury, or
    2) resulted in a permanent impairment of a body structure or a body function, or
    3) required in-patient or prolongation of hospitalization, or
    4) resulted in medical or surgical intervention to prevent life-threatening illness or injury or permanent impairment to body structure or a body function
c) Led to fetal distress, fetal death or a congenital abnormality or birth defect.

NOTE: Planned hospitalization for a pre-existing condition, or a procedure required by the protocol, without serious deterioration in health, is not considered a serious adverse event.

8.1.3 Unanticipated Adverse Device Effect (UADE/USADE)

An Unanticipated ADE (UADE) is any serious adverse effect on health or safety or any life-threatening problem or death caused by or associated with a device, if that effect, problem, or death was not previously identified in nature, severity, or degree of incidence in the protocol or application (including a supplementary plan or application (e.g., user manual, investigator's brochure, instructions for use, etc.)); or any other unanticipated serious problem associated with a device that relates to the rights, safety, or welfare of subjects (ISO14155:2011E).

8.1.4 Device Deficiency, Device Malfunction, and Use Error

Device Deficiency

Defined as inadequacy of a medical device with respect to its identity, quality, durability, reliability, safety, or performance

Device Malfunction

Defined as a failure of a medical device to perform in accordance with its intended purpose when used in accordance with the instructions for use or protocol or user manual.

Use error

Defined as the act or omission of an act that results in a different medical device response than intended by the manufacturer or expected by the user.

- Note 1: Use error includes slips, lapses, and mistakes.

- Note 2: An unexpected physiological response of the subject does not in itself constitute a use error.

Device deficiencies are also reportable if they did not lead to an adverse event but could have led to a SADE:

a) if either suitable action had not been taken,
b) if intervention had not been made, or
c) if circumstances had been less fortunate

(ISO14155:2011E)

8.2 Reporting Device and/or Procedure Related Adverse Events

All suspected device related adverse events shall be recorded on the Adverse Event page of the CRF if they are suspected to be related to the use of the NeuralBot Investigational System. If appropriate, the event shall subsequently be reported to the relevant IRB. The event shall be thoroughly investigated and a causal relation as to whether the event is related or not to the use of the NeuralBot Investigational System shall be established.

The Investigator will record the nature, severity, relatedness, treatment, and outcome of the AE. This classification of the event determines the reporting procedures to be followed. NovaSignal may upgrade the classification as required for reporting purposes.

At the initiation of device use through end of use of the device, all subjects with adequate quality NeuralBot Investigational System data shall be assessed for any potentially device related complications or adverse events. All events shall be followed until resolution or through the end of a subject's study participation.

Device-related adverse events information will be collected throughout the study. From the initiation of and end of device use, all subjects scanned with the NeuralBot Investigational System shall be assessed for any potentially device related complications or adverse events. All events shall be followed until resolution or through the end of a subject's study participation.

Procedure-related adverse events related to agitated saline injection before and after VM (headache, allergic reactions, new onset neurological deficit, ischemic stroke, TIA or pulmonary embolism complicating agitated saline contrast injection, etc) will be closely monitored and prospectively collected[18]. Nevertheless, it should be noted that a recent multi-center study underlined the safety and importance in performing TCD-BS for RLS detection in a standardized manner using the currently recommended international consensus protocol with agitated saline intravenous injections[19].

Event, onset date, severity, relatedness, device relationship, treatment and outcome of the AE will be recorded on the appropriate case report form. Any device-related and/or procedure-related AEs will be monitored until they are adequately resolved or explained. This classification of the event determines the reporting procedures to be followed. For purposes of this protocol, the following definitions will apply.

8.3 Classification of a Device and/or Procedure Related Adverse Event

8.3.1 Severity Rating of Event

The following categories of adverse event severity are to be used:

**Mild**	Awareness of sign or symptom that does not interfere with the subject's usual activity or is transient, resolved without treatment and with no sequelae.
**Moderate**	Interferes, but does not hinder, the subject's usual activity and/or may require treatment.
**Severe**	Symptom(s) causing severe discomfort and significant impact on the subject's usual activity and requires treatment or intervention.

8.3.2 Relationship of Study Device and/or Procedure

The causal relationship to study device and/or procedure will be evaluated as follows:

**Caused By**	Relation	Definition of Relation
**Study Device**	**Definitely Related**	The event is clearly related to the study device beyond reasonable doubt.
	**Probably Related**	The event is temporally associated and plausibly related to the study device but there are also potential alternative explanations, though the alternatives are not likely.
	**Possibly Related**	The adverse event may be related, scientifically plausible to the study device, but there are also alternative explanations.
**Procedure**	**Definitely Related**	The event is clearly related to the procedure beyond reasonable doubt.
	**Probably Related**	The event is temporally associated and plausibly related to the procedure but there are also potential alternative explanations, though the alternatives are not likely.
	**Possibly Related**	The adverse event may be related, scientifically plausible to the procedure, but there are also alternative explanations.

8.3.3 Outcome of Event

The outcome of each Device and/or Procedure Related Adverse Event must be assessed according to the following classifications:

**Classification**	**Definition**
**Resolved**	Subject fully recovered with no observable residual effects
**Resolving**	Subject's condition improved, but residual effects remain

**Resolved with Sequelae**	Subject recovered with observable residual effects
**Ongoing**	Event is ongoing without changes in the overall condition
**Fatal**	Subject died as a result of the device and/or procedure related event

8.4 Reporting Procedures

All UADE/SADE/SAEs must be recorded and reported to the Sponsor via the eCRFs throughout the study, ***immediately upon study site staff awareness of the event but not later than 72 hours by the study site personnel.*** In the event the EDC is unavailable, events can be reported via email to the Sponsor study mailbox: events@novasignal.com and/or to the study Sponsor contact. Note: The event will still need to be recorded on the eCRFs once the EDC is functional.

In case device malfunctions occur, they will be reported to NovaSignal within 10 business days. The report should include at a minimum, a description of event, date of occurrence, lot or serial number of the device.

Timing for the reporting for the different types of AEs and Device Deficiencies is described as follows:

Classification	Reporting time	Type of report
**Unanticipated Adverse Device Effects (UADE)**	Notify NovaSignal immediately upon study site staff awareness of event but not later than 72 hours. Notify IRB as required.	Device/Procedure Related Serious Adverse Event Report Form
**Serious Adverse Events (SAE) or Serious Adverse Device Effect (SADE)**	Notify NovaSignal immediately upon study site staff awareness of event but not later than 72 hours. Notify IRB as required.	Device/Procedure Related Serious Adverse Event Report Form
**Study Device Deficiency/Malfunction With or Without AE**	Notify NovaSignal within 10 business days of learning of event. Notify IRB as required.	Device Deficiency/ Malfunction Form

9 PROTOCOL DEVIATIONS

A protocol deviation is defined as a failure to comply (intentionally or unintentionally) with the requirements of the clinical study as specified in the protocol.

Examples of deviations include but are not limited to a required test not being done or not being done within the specified timeframe, a subject enrolled who did not meet the inclusion/exclusion criteria, or enrolment of a subject without appropriate consent.

Deviations from the protocol must be reported to NovaSignal through the eCRFs and will be reviewed and assessed by the Sponsor.

The site is responsible to use vigilance to identify and report deviations to the Sponsor and per site IRB policies. The study monitors shall verify that the conduct of the study is in compliance with the approved protocol and applicable regulations and shall identify deviations and any issues of noncompliance. Corrective and preventative actions will be implemented promptly and as necessary. Significant protocol deviations that raise subject safety concerns or indicate repeat noncompliance may be grounds for investigator disqualification.

The Investigator must provide reports of the progress, completion, termination or discontinuation of the study to the IRB(s) at appropriate intervals as designated by the Sponsor and per IRB requirements.

10 QUALITY CONTROL AND QUALITY ASSURANCE

10.1 Organizational Preparations

A site evaluation via personnel and facility documentation will be performed by NovaSignal or their designee during the study to ensure the availability of appropriately trained personnel to conduct the study according to the FDA Code of Federal Regulations and ICH Guidelines on Good Clinical Practices (ICH-E6). This study will be conducted under the principles described in the Declaration of Helsinki.

10.2 Training

NovaSignal will provide training for the use of the NeuralBot Investigational System, in adherence to the Instructions for Use/User Manual. Documentation of training should be maintained by the Principal Investigator throughout the study.

10.3 Data Quality Assurance

Training of appropriate site personnel will be the responsibility of NovaSignal or designee. To ensure uniform data collection and protocol compliance, site personnel will utilize source documentation worksheets to document protocol procedures. Principal investigators will assist in developing training modules for standardization of clinical technique.

10.4 Subject Privacy

All data will be maintained under highly secure and fully HIPAA-compliant dedicated servers under the direction supervision of the Principal Investigator. Primary data that is obtained will have identifying information and will be stored securely onsite. Access to identifiable subject data is limited to project staff who have direct data management and/or statistical responsibilities, under the direct supervision of the Principal Investigator.

The study participant’s contact information will be securely stored at each clinical site for internal use during the study. At the end of the study, all records will continue to be kept in a secure location for as long a period as dictated by the reviewing IRB.

Data de-identification will be performed manually using the "safe-harbor" approach under the supervision of the Principal Investigator. At this point, a unique ID is generated for each subject, and each exam. These will populate two databases using the "safe-harbor" approach under the supervision of the Principal Investigator. The database that is devoid of all PHI will be used for data analysis in this research.

10.5 Case Report Forms

All required data for this study will be collected on standardized Case Report Forms (CRF). The forms may be paper based or electronic. The forms will only include the subject study number assigned once the subject is screened. The investigator is responsible for the accuracy, completeness and legibility of the data reported to NovaSignal in the CRFs and in all required reports. The CRFs are to be dated and signed by an investigator on appropriate pages to verify that he/she has reviewed the recorded data.

10.6 Investigational Device Accountability

The NeuralBot Investigational System specific serial numbers must be documented at a study site by a designated person, handled and stored properly in a secured location in which only the study staff have access. The Principal Investigator must maintain an accurate record of the status of the products throughout the study. Investigators are responsible for appropriate logging of the devices used, verification of packing slip information (i.e. lot numbers and quantity shipped), date and identity that each device was used in the study, disposition information regarding disposal or return to the Sponsor.

10.7 Selection of Investigators

The study will be conducted at multiple centers. Study staff for each center will meet the following criteria:

- A user trained by NovaSignal in the use of the Lucid M1 System and NeuralBot Investigational System.
- Commitment from the participating investigator to pursue details of any potentially device and procedure related adverse event outcomes.
- Commitment from the participating investigator to enroll only subjects meeting inclusion and exclusion criteria of the local approved protocol.
- Dedicated staff members who can collect and transmit data and be willing to maintain all study-related documentation (e.g., source documents, CRFs, regulatory documentation, etc.)

10.8 Close-out Document Review

The purpose of the final document review is to collect all outstanding study data documents, ensure that the principal investigator's files are accurate and complete, review record retention requirements with the principal investigator and ensure that all applicable requirements are met for the study.

The investigator agrees to allow the monitoring of study data, the completion of all data clarification or audits even after study close-out visit has been performed at NovaSignal's request.

11 STATISTICAL ANALYSIS

11.1 Primary Endpoint Analysis

- Demonstration of NB-IS TCD diagnostic performance for shunt detection, as compared to transthoracic echocardiography (TTE) for diagnostic accuracy.
    - Diagnostic Accuracy - Shunt detection rate (%)

- % detection of true positives (TP) (sensitivity), all sample size estimations will be computed with regard to the endpoint of sensitivity

11.2 Secondary Technical Efficacy Endpoint(s) Analysis

- % detection of false positives (FP), comparison of NB-IS TCD with TTE
- % detection of true negatives (TN) (specificity), comparison of NB-IS TCD with TTE
- % detection of false negatives (FN), comparison of NB-IS TCD with TTE
- Positive Predictive Value (PPV), comparison of NB-IS TCD with TTE
- Negative Predictive Value (NPV), comparison of NB-IS TCD with TTE

- Demonstration of NB-IS TCD diagnostic performance to SOC TCD for diagnostic accuracy parameters (sensitivity, specificity, PPV, NPV).
  - If bilateral signals are found, the microbubble count will be sum across both vessels.
  - If only a unilateral signal is found, the microbubble count will be doubled for the total count.
  - Spencer Logarithmic Scale (SLS) and International Consensus Criteria (ICC) gradings from the Imaging Core Lab for NB-IS TCD and SOC TCD will be compared for analysis.

- Demonstration of NB-IS TCD diagnostic performance targeting to transesophageal echocardiography (TEE) for diagnostic accuracy parameters (sensitivity, specificity, PPV, NPV).

- % detection of intervenable shunts, comparison of NB-IS TCD with TTE
  - For NB-IS TCD, Grade 3 and above (>30 microbubbles) on the Spencer Logarithmic Scale (SLS) and International Consensus Criteria (ICC) considered intervenable shunt[10].
  - For TTE, >20 microbubbles per 2016 American Society of Echocardiography Guidelines and Standards[23] considered intervenable shunt.

- NB-IS TCD No Window rate performance to SOC TCD parameters (including both unilateral and bilateral absent acoustic windows).

- % success rate of NB-IS TCD
  - Incidence of failed registrations (device unable to register)
  - Incidence of bilateral signals not found (no evaluable signals found both sides)
  - Incidence of unilateral signals not found (evaluable signal found on one side)
  - An adequate, evaluable, and complete study is defined for the purposes of this protocol as successful acquisition of a unilateral signal at 40-65mm depth range recorded for the duration of the bubble study in the Resting position and with Valsalva Maneuver.

- Incidence of device malfunctions

11.3 Exploratory Endpoints Analysis

- Demonstration of NB-IS TCD accuracy to Spencer Logarithmic Scale (SLS) Grade and International Consensus Criteria (ICC) grade for diagnostic accuracy.

11.4 Imaging Core Lab Analysis

All de-identified imaging data will be sent to a core laboratory which will provide independent quantitative and qualitative assessment of all NB-IS TCD and SOC TCD, TTE, and TEE bubble study data. They will be blinded to the study and local diagnostic report data and provide independent review. The Core Lab interpretations will supersede all local interpretations and will be applied to all study endpoint analyses as applicable.

11.5 RLS/PFO Validation and Analysis

Evaluation of correlation between diagnostic imaging standard of care (SOC TCD/TTE/TEE) and NB-IS TCD diagnostic performance by an independent core laboratory. Success criteria will include clinical validation of NB-IS TCD for RLS/PFO detection targeting (i) 40% increase in NB-IS TCD sensitivity compared to TTE sensitivity and (ii) NB-IS TCD sensitivity ≥ 90% compared to SOC TCD sensitivity.

11.6 Sample Size Calculation

The present study was powered based on the results of a recent meta-analysis reporting a pooled TCD sensitivity of 96.1% for RLS detection, while the pooled TTE sensitivity was estimated at 45.1% (absolute difference of 51%)[6]. For power calculations, we used a more moderate effect size of 40% increase in the sensitivity of NB-IS TCD compared to TTE.

A sample size of 100 subjects achieves 90% power to detect a difference of 40% between two diagnostic tests whose sensitivities are 90% (TCD) and 50% (TTE). This procedure uses a two-sided McNemar test with a significance level of 0.05. The mean prevalence of PFO in the population of patients with cryptogenic stroke is at least 30%[2]. The proportion of discordant pairs has been set at 0.500.

Power	N	Sensitivity of Test 1 Se1	Sensitivity of Test 2 Se2	Difference Se1-Se2	Proportion Discordant D	Prevalence P	Alpha	Beta
0.00000	10	0.900	0.500	0.400	0.500	0.300	0.05000	1.00000
0.00830	20	0.900	0.500	0.400	0.500	0.300	0.05000	0.99170
0.12990	30	0.900	0.500	0.400	0.500	0.300	0.05000	0.87010
0.32013	40	0.900	0.500	0.400	0.500	0.300	0.05000	0.67987
0.49003	50	0.900	0.500	0.400	0.500	0.300	0.05000	0.50997
0.62345	60	0.900	0.500	0.400	0.500	0.300	0.05000	0.37655
0.72404	70	0.900	0.500	0.400	0.500	0.300	0.05000	0.27596
0.79937	80	0.900	0.500	0.400	0.500	0.300	0.05000	0.20063
0.85654	90	0.900	0.500	0.400	0.500	0.300	0.05000	0.14346
0.89995	100	0.900	0.500	0.400	0.500	0.300	0.05000	0.10005
0.93182	110	0.900	0.500	0.400	0.500	0.300	0.05000	

	0.06818						
0.95405	120	0.900	0.500	0.400	0.500	0.300	0.05000
	0.04595						
0.96898	130	0.900	0.500	0.400	0.500	0.300	0.05000
	0.03102						
0.97890	140	0.900	0.500	0.400	0.500	0.300	0.05000
	0.02110						
0.98561	150	0.900	0.500	0.400	0.500	0.300	0.05000
	0.01439						

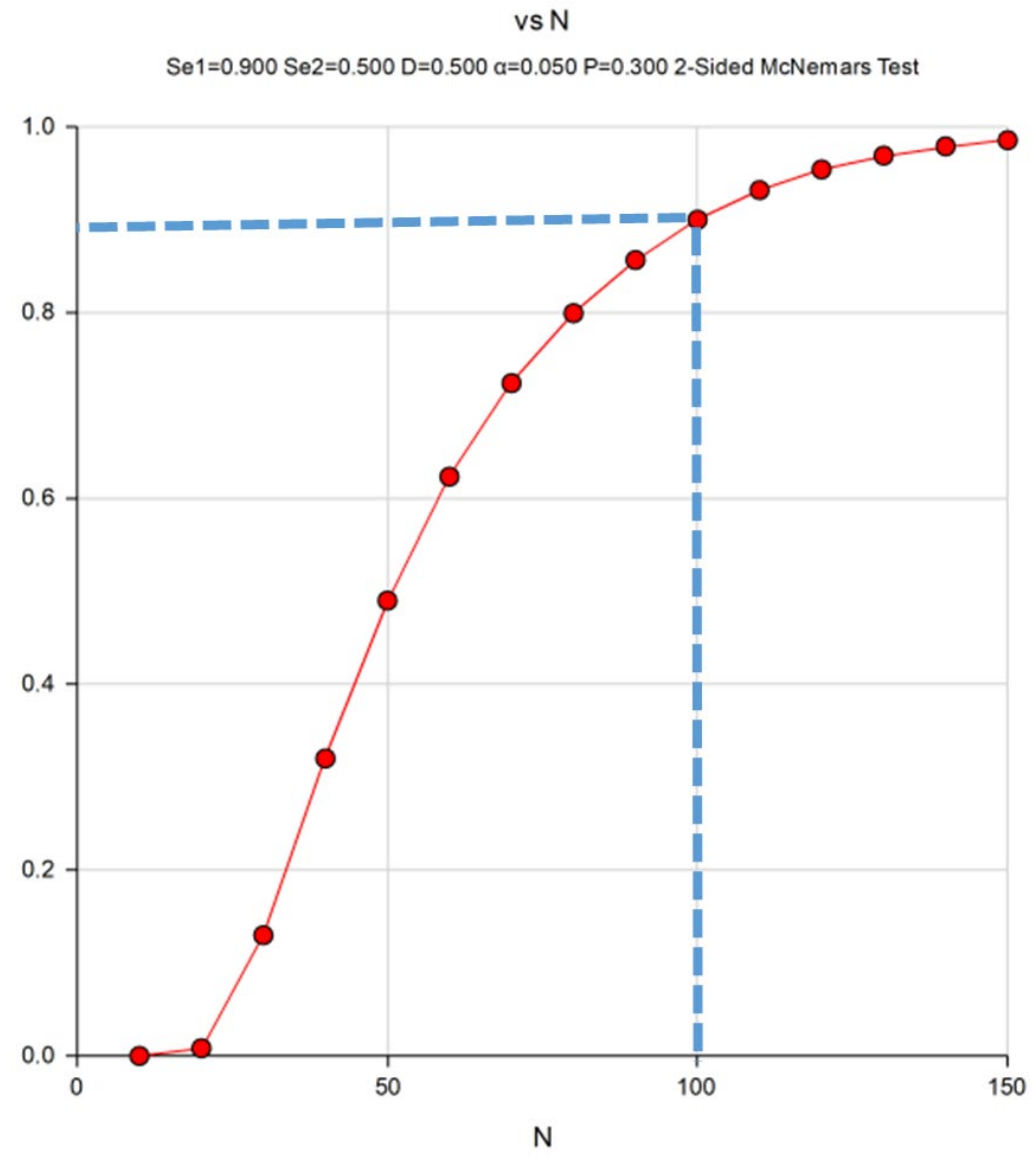

Given previous reports indicating a prevalence of suboptimal transtemporal windows in 5% of Hispanic[20], 5% of Caucasian[21], 9% in African American[19] and 14% of Asian[22] individuals aged <60 years, we increased our projected sample size by 20% (n=120). In addition, the final sample size

was further increased by 20% (n=144) in order to account for an anticipated dropout rate of 20%. Consequently, the final study sample was set at 144 individuals.

The sensitivity of TCD against TEE for right-to-left shunt detection has been estimated 90% in a recent meta-analysis[24]. A sample size of 147 patients produces a two-sided 95%CI with a 95%CI width equal to 20% when the sample sensitivity is 90% and the prevalence is 30%. After the first 150 TTE subjects in the Main study are enrolled, the study will continue to enroll up to an additional 150 subjects that received a TTE or TEE bubble study.

11.7 Primary Safety Endpoint Analysis

Throughout the study we will collect the incidence of device-related adverse events, which will be used to determine the safety of the device within the acute stroke environment. In our EXPEDITE study which is also run in the ER for stroke assessment, the device has been designated minimal risk by the IRB and we have not had any serious adverse events in over 80 subjects. We will continue to closely monitor this metric.

11.8 Secondary Safety Endpoint Analysis

Throughout the study we will collect the incidence of procedure-related adverse events related to agitated saline injection before and after VM (headache, allergic reactions, new onset neurological deficit, ischemic stroke, TIA or pulmonary embolism complicating agitated saline contrast injection, etc.) will be closely monitored and prospectively collected[18].

12 ETHICAL CONSIDERATIONS

12.1 Investigational Review Board (IRB) Approval

This study will be conducted using an FDA cleared product called the Lucid M1 System, 510k K160442. The study will also utilize an investigational device, called the NeuralBot Investigational System (NB-IS) 510k K180455 with modifications to facilitate workflow improvements in the study. The modifications do not introduce additional risks and do not alter existing risks and mitigations. All modifications will be tested per NovaSignal's design controls procedures.

This protocol and the informed consent must be reviewed and approved by the appropriate IRB where the study is to be conducted before enrolment of subjects. The NovaSignal and the IRB must approve in writing any changes to the protocol that affect the rights, safety, and/or welfare of the subjects, or may adversely affect the validity of the study.

It is the responsibility of the investigator to submit the final version of the protocol with the Informed Consent Form (ICF), if required, to an appropriately constituted IRB prior to commencement of the study. The Investigator will submit the appropriate documentation if any extension, renewal or amendment of the IRB approval must be obtained. In particular, study plan amendments, ICF changes or other written information provided to the subject must be approved by the IRB in writing, when required.

12.2 Role of NovaSignal

As the study Sponsor of this clinical study, NovaSignal has the overall responsibility for the conduct of the study, including assurance that the study meets the regulatory requirements of the Food and Drug Administration. NovaSignal will ensure adherence to the regulations as outlined in

the Sponsor general duties, selection of investigators, monitoring, maintaining records, and submitting reports.

12.3 Investigator Responsibilities

The Principal Investigator (PI) shall be responsible for the day-to-day conduct of the study as well as for the safety and well-being of the human subjects involved in the study. The PI also assumes overall responsibility and accountability for the study team and for data obtained from each subject participating in the study.

The PI shall be responsible for:

1. Obtaining a written IRB approval for the study and subject ICF prior to including any subject in this study, as required by local rules and laws.
2. Ensuring that the study is conducted in compliance with IRB requirements including conditions which may be imposed by a reviewing IRB.
3. Ensuring compliance with the study plan, applicable laws, and applicable regulations.
4. Obtaining informed consent and privacy authorization for all study subjects prior to subject participation (the informed consent and privacy authorization processes may be combined, as per usual procedure of the IRB).
5. Collecting all required study data on the Case Report Forms provided.
6. Reviewing and signing CRF pages indicating documents are accurate and complete.

The PI will agree to provide access to the records of all subjects entered into this study, as well as all other study documentation. In addition, all records may be subject to inspection by officials of US FDA and other regulatory authorities according to local rules and laws.

The PI should make accurate and adequate progress reports to the IRB at appropriate intervals, according to the IRB requirements, when applicable. The PI will inform the IRB of study completion or termination within the time period specified by the IRB, when applicable.

The PI is responsible for informing the IRB of any safety issues related to the study as required.

The PI/site must maintain adequate records on all aspects of the study.

The PI/site must maintain the study records for at least two years after cessation of the study.

Sub-Investigators will be responsible for study activities in coordination with the PI and in accordance to the study plan. A Sub-Investigator may assume the responsibility of the PI should the PI resign from the study.

12.4 Records Custody

An investigator may withdraw from the study. If the PI withdraws from the study, the responsibility of conducting follow-up and maintaining records must be transferred to another responsible party within institution (i.e. Sub-I). Notice of transfer must be provided in writing by the PI to NovaSignal and the IRB when applicable, not later than 10 working days after transfer occurs.

13 APPENDIX

13.1 Spencer Logarithmic Scale (SLS)[10]

SLS GRADE	SLS Microembolic Signal (MES) Count
Grade 0	No MES
Grade 1	1 – 10 MES
Grade 2	11 – 30 MES
Grade 3	31 – 100 MES
Grade 4	101 – 300 MES
Grade 5	> 300 MES

13.2 International Consensus Criteria (ICC)[11]

ICC GRADE	ICC Microembolic Signal (MES) Count
Grade 0	No MES
Grade 1	1 – 20 MES
Grade 2	> 20 MES or “shower” appearance
Grade 3	“curtain” appearance of MES

13.3 Classification of Potential Causative Mechanism in PFO–Associated Stroke[1]

		RoPE Score	
**Risk Source**	**Features**	**Low**	**High**
Very High	A PFO and a straddling thrombus	Definite	Definite
High	(1) Concomitant pulmonary embolism or deep venous thrombosis preceding an index infarct combined with either (2a) a PFO and an atrial septal aneurysm or (2b) a large-shunt PFO	Probable	Highly probable
Medium	Either (1) a PFO and an atrial septal aneurysm or (2) a large-shunt PFO	Possible	Probable
Low	A small-shunt PFO without and atrial septal aneurysm	Unlikely	Possible

14 REFERENCES

1. Elgendy AY, Saver JL, Amin Z, Boudoulas KD, Carroll JD, Elgendy IY, Grunwald IQ, Gertz ZM, Hijazi ZM, Horlick EM, Kasner SE, Kent DM, Kumar P, Kavinsky CJ, Liebeskind DS, Lutsep H, Mojadidi MK, Messé SR, Mas JL, Mattle HP, Meier B, Mahmoud A, Mahmoud AN, Nietlispach F, Patel NK, Rhodes JF, Reisman M, Sommer RJ, Sievert H, Søndergaard L, Zaman MO, Thaler D, Tobis JM; MSCAI14. Proposal for Updated Nomenclature and Classification of Potential Causative Mechanism in Patent Foramen Ovale-Associated Stroke. JAMA Neurol. 2020 Apr 13. doi:10.1001/jamaneurol.2020.0458. [Epub ahead of print] PubMed PMID: 32282016.

2. Katsanos AH, Giannopoulos S, Frogoudaki A, Vrettou AR, Ikonomidis I, Paraskevaidis I, Zompola C, Vadikolias K, Boviatsis E, Parissis J, Voumvourakis, K, Kyritsis AP, Tsivgoulis G. The diagnostic yield of transesophageal echocardiography in patients with cryptogenic cerebral ischaemia: a meta-analysis. Eur J Neurol. 2016 Mar;23(3):569-79.

3. Mojadidi M, Zaman M. Cryptogenic stroke and patent foramen ovale. *Am Coll Cardiol*. 2018;89(10):1143-1153. doi:10.1007/s00115-018-0609-y

4. Hart RG, Diener HC, Coutts SB, Easton JD, Granger CB, O'Donnell MJ, et al.; Cryptogenic Stroke/ESUS International Working Group. Embolic strokes of undetermined source: the case for a new clinical construct. Lancet Neurol. 2014; 13:429–438

5. Pristipino C, Sievert H, D'Ascenzo F, Louis Mas J, Meier B, Scacciatella P, Hildick-Smith D, Gaita F, Toni D, Kyrle P, Thomson J, Derumeaux G, Onorato E, Sibbing D, Germonpré P, Berti S, Chessa M, Bedogni F, et al. European position paper on the management of patients with patent foramen ovale. General approach and left circulation thromboembolism. *Eur Heart J*. 2019;40(38):3182-3195. doi:10.1093/eurheartj/ehy649

6. Katsanos AH, Psaltopoulou T, Sergentanis TN, Frogoudaki A, Vrettou AR, Ikonomidis I, Paraskevaidis I, Parissis J, Bogiatzi C, Zompola C, Ellul J, Triantafyllou N, Voumvourakis K, Kyritsis AP, Giannopoulos S, Alexandrov AW, Alexandrov A V., Tsivgoulis G. Transcranial Doppler versus transthoracic echocardiography for the detection of patent foramen ovale in patients with cryptogenic cerebral ischemia: A systematic review and diagnostic test accuracy meta-analysis. *Ann Neurol*. 2016;79(4):625-635. doi:10.1002/ana.24609

7. Mojadidi MK, Winoker JS, Roberts SC, Msaouel P, Zaman MO, Gevorgyan R, Tobis JM. Accuracy of conventional transthoracic echocardiography for the diagnosis of intracardiac right-to-left shunt: A meta-analysis of prospective studies. *Echocardiography*. 2014;31(9):1036-1048. doi:10.1111/echo.12583

8. Sarkar S, Ghosh S, Ghosh SK, Collier A. Role of transcranial Doppler ultrasonography in stroke. *Postgrad Med J*. 2007;83(985):683-689. doi:10.1136/pgmj.2007.058602

9. Alexandrov AV, Sloan MA, Tegeler CH, Newell DN, Lumsden A, Garami Z, Levy CR, Wong LK, Douville C, Kaps M, Tsivgoulis G; American Society of Neuroimaging Practice Guidelines Committee. Practice standards for transcranial Doppler (TCD) ultrasound. Part II. Clinical indications and expected outcomes. J Neuroimaging. 2012 Jul;22(3):215-24.

10. Spencer MP, Moehring MA, Jesurum J, Gray WA, Olsen JV, Reisman M. Power M mode transcranial doppler for diagnosis of patent foramen ovale and assessing transcatheter closure. J Neuroimaging. 2004;14:342-349.

11. Jauss M, Zanette E. Detection of right-to-left shunt with ultrasound contrast agent and transcranial Doppler sonography. Cerebrovasc Dis 2000;10:490-496.

12. Romero JR, Frey JL, Schwamm LH, et al. Cerebral ischemic events associated with “bubble study” for identification of right to left shunts. Stroke 2009;40:2343-2348

13. Hart RG, Diener HC, Coutts SB, Easton JD, Granger CB, O’Donnell MJ, et al.; Cryptogenic Stroke/ESUS International Working Group. Embolic strokes of undetermined source: the case for a new clinical construct.Lancet Neurol. 2014;13:429–438.

14. Sastry S, Daly, K, Chengodu, T and McCollum, C. Is transcranial Doppler for the detection of venous-to-arterial circulation shunts reproducible? Cerebrovasc Dis. 2007; 5-6:424-429.

15. Gentile M, De Vito A, Azzini C, Tamborino C, Casetta I. Adding blood to agitated saline significantly improves detection of right-to-left shunt by contrast-transcranial color-coded duplex sonography. Ultrasound Med Biol. 2014 Nov;40(11):2637-41.

16. Malhotra K, Katsanos AH, Frogoudaki A, Vrettou A, Ikonomidis I, Tsivgoulis G, Patent Foramen Ovale: Risk Factors and Diagnostic Comparison of Transcranial Doppler versus Echocardiography for Secondary Stroke Prevention. J Neurosonol Neuroimag. 2019;11:22-33

17. Alexandrov AV, Sloan MA, Tegeler CH, Newell DN, Lumsden A, Garami Z, Levy CR, Wong LK, Douville C, Kaps M, Tsivgoulis G; American Society of Neuroimaging Practice Guidelines Committee. Practice standards for transcranial Doppler (TCD) ultrasound. Part II. Clinical indications and expected outcomes. J Neuroimaging. 2012 Jul;22(3):215-24.

18. Romero JR, Frey JL, Schwamm LH, et al. Cerebral ischemic events associated with “bubble study” for identification of right to left shunts. Stroke 2009;40:2343-2348.

19. Tsivgoulis G, Stamboulis E, Sharma VK, Heliopoulos I, Voumvourakis K, Teoh HL, Vadikolias K, Triantafyllou N, Chan BP, Vasdekis SN, Piperidou C. Safety of transcranial Doppler 'bubble study' for identification of right to left shunts: an international multicentre study. J Neurol Neurosurg Psychiatry. 2011Nov;82(11):1206-8.

20. Brunser AM, Silva C, Cárcamo D, Muñoz P, Hoppe A, Olavarría VV, Díaz V, Abarca J. Transcranial Doppler in a Hispanic-Mestizo population with neurological diseases: a study of sonographic window and its determinants. Brain Behav. 2012 May;2(3):231-6.

21. Marinoni M, Ginanneschi A, Forleo P, Amaducci L. Technical limits in transcranial Doppler recording: inadequate acoustic windows. Ultrasound Med Biol. 1997;23(8):1275-7.

22. Itoh T, Matsumoto M, Handa N, Maeda H, Hougaku H, Hashimoto H, Etani H, Tsukamoto Y, Kamada T. Rate of successful recording of blood flow signals in the middle cerebral artery using transcranial Doppler sonography. Stroke. 1993 Aug;24(8):1192-5.

23. Saric M, Armour AC, Arnaout MS, et al. Guidelines for the Use of Echocardiography in the Evaluation of a Cardiac Source of Embolism. *J Am Soc Echocardiogr*. 2016;29(1):1-42. doi:10.1016/j.echo.2015.09.011.

24. Katsanos AH, Psaltopoulou T, Sergentanis TN, et al. Transcranial Doppler versus transthoracic echocardiography for the detection of patent foramen ovale in patients with cryptogenic cerebral ischemia: A systematic review and diagnostic test accuracy meta-analysis. Ann Neurol. 2016;79(4):625-635

RoBotic TCD Ultrasound BubbLe Study Compared to Transthoracic Echocardiography for Detection of Right to Left Shunt

BUBL Statistical Analysis Plan

Document Revision History:

Version	Date	Content/Modifications
01	21MAY2020	Original Document
02	15JUN2020	Amendment to add exclusion criteria #2 for pregnant women per WIRB recommendation
03	11AUG2020	Administrative change from Neural Analytics to NovaSignal company name change
04	12FEB2021	Removed upper limit of age Inclusion Criteria.<br>Added continuation of enrollment after the main study subjects with TTE are enrolled up to another 150 subjects with TEE performed
05	02AUG2021	Revised Sub-Study to include TTE or TEE after Main Study subjects are enrolled.<br>Removed single-site sub-study at UTHSC.
06	29JUN2022	Biostatistical methodology addition

Table of Contents

1 Abbreviations 3
2 Sample Size Justification 3
3 Study Populations 5
4 Statistical Methods 5
4.1 General Reporting Conventions 5
4.2 Adjustments for Interim Analyses and Multiplicity of Endpoints 5
4.3 Evaluations and Statistical Analyses 5
4.3.1 Subject Disposition 5
4.3.2 Demographics and Baseline Characteristics 5
4.3.3 Primary Efficacy Outcome 5
4.3.3.1 Primary Efficacy Hypothesis 5
1. 6
4.3.3.2 Primary Efficacy Analysis 6
4.3.3.3 Sensitivity and Supporting Analyses 6
4.3.4 Secondary Efficacy Outcomes 6
4.3.5 Exploratory Analysis Endpoint Analyses 7
5 List of Tables, Figures and Listings 7

1 Abbreviations

AE	Adverse Event
DM	Device Malfunction
IRB	Institutional Review Board
ITT	Intent-to-Treat
PFO	Patent Foramen Ovale
PP	Per-Protocol
raTCD	Robot-Assisted Transcranial Doppler
RLS	Right to Left Shunt
SAE	Serious Adverse Event
SOC	Standard of Care
UADE	Unanticipated Adverse Device Effects
TCD	Transcranial Doppler
TEE	Transesophageal Echocardiograph
TTE	Transthoracic Echocardiograph

2 Sample Size Justification

Table 1: Sample Size and Power Assessment

Power	N	Sensitivity of Test 1 Se1	Sensitivity of Test 2 Se2	Proportion Difference Se1-Se2	Discordant D	Prevalence P	Alpha	Beta
0.00000	10	0.900	0.500	0.400	0.500	0.300	0.05000	1.00000
0.00830	20	0.900	0.500	0.400	0.500	0.300	0.05000	0.99170
0.12990	30	0.900	0.500	0.400	0.500	0.300	0.05000	0.87010
0.32013	40	0.900	0.500	0.400	0.500	0.300	0.05000	0.67987
0.49003	50	0.900	0.500	0.400	0.500	0.300	0.05000	0.50997
0.62345	60	0.900	0.500	0.400	0.500	0.300	0.05000	0.37655
0.72404	70	0.900	0.500	0.400	0.500	0.300	0.05000	0.27596
0.79937	80	0.900	0.500	0.400	0.500	0.300	0.05000	0.20063
0.85654	90	0.900	0.500	0.400	0.500	0.300	0.05000	0.14346
0.89995	100	0.900	0.500	0.400	0.500	0.300	0.05000	0.10005
0.93182	110	0.900	0.500	0.400	0.500	0.300	0.05000	0.06818
0.95405	120	0.900	0.500	0.400	0.500	0.300	0.05000	0.04595
0.96898	130	0.900	0.500	0.400	0.500	0.300	0.05000	0.03102
0.97890	140	0.900	0.500	0.400	0.500	0.300	0.05000	0.02110

The present study was powered based on the results of a recent meta-analysis reporting a pooled TCD sensitivity of 96.1% for RLS detection, while the pooled TTE sensitivity was estimated at 45.1% (absolute difference of 51%). For power calculations, we used a more moderate effect size of 40% increase in the sensitivity of raTCD compared to TTE.

A sample size of 100 subjects achieves 90% power to detect a difference of 40% between two diagnostic tests whose sensitivities are 90% (TCD) and 50% (TTE). This procedure uses a two- sided McNemar test with a significance level of 0.05. The mean prevalence of PFO in the population of patients with cryptogenic stroke is at least 30%. The proportion of discordant pairs has been set at 0.500.

Figure 1: Sample Size and Power Assessment

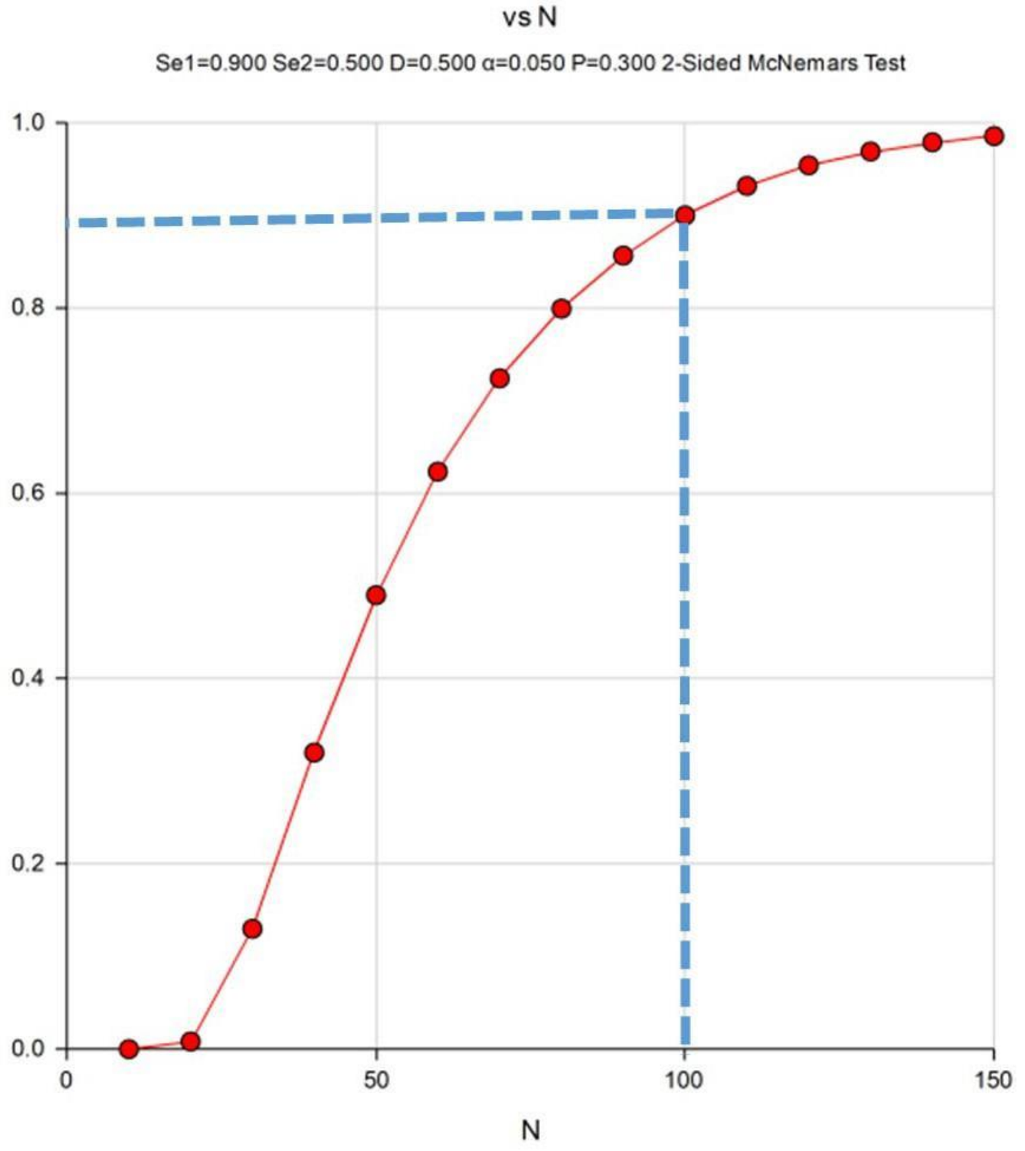

Given previous reports indicating a prevalence of suboptimal transtemporal windows in 5% of Hispanic, 5% of Caucasian, 9% in African American and 14% of Asian individuals aged <60 years, we increased our projected sample size by 20% (n=120). In addition, the final sample size was further increased by 20% (n=144) in order to account for an anticipated dropout rate of 20%. Consequently, the final study sample was set at 144 individuals.

The sensitivity of TCD against TEE for right-to-left shunt detection has been estimated 90% in a recent meta-analysis. A sample size of 147 patients produces a two-sided 95%CI with a 95%CI width equal to 20% when the sample sensitivity is 90% and the prevalence is 30%.

3 Study Populations

Study Analysis Populations

Population	Definition
Intent-to-Scan (ITS)	Defined as subjects that met all Inclusion/Exclusion criteria and raTCD was attempted.
Per-Protocol	Defined as subjects that met all Inclusion/Exclusion criteria and successfully completed the study with no protocol deviations and complete data sets.

The ITS population will be used for the primary outcome and the PP population will be used for secondary outcomes.

4 Statistical Methods

4.1 General Reporting Conventions

Study data will be provided in listings and sorted by subject number, study period, and assessment time. Standard numeric descriptive statistics include number of subjects or records observed (N), mean, standard deviation (std), median, minimum (min), and maximum (max) values. Standard categorical descriptive statistics include the count and percentages of subjects with a level of the variable summarized. Two-sided p-values of less than or equal to 0.05 will be considered statistically significant.

4.2 Adjustments for Interim Analyses and Multiplicity of Endpoints

The study includes a single primary effectiveness outcome, there are no planned interim analyses; hence, no adjustment for multiplicity will be implemented.

4.3 Evaluations and Statistical Analyses

4.3.1 Subject Disposition

Of the subjects that were eligible, the number of subjects that were enrolled and the number of subjects that completed the study will be summarized. The number and percentages for reasons that subjects did not enroll or complete the study will also be summarized.

4.3.2 Demographics and Baseline Characteristics

Demographics information will be summarized using descriptive statistics. For gender, race, and ethnicity the summarization of those categorical values will be performed using percentages and frequency. Age will be summarized using descriptive statistics as total number of subjects surveyed (N), mean, standard deviation [SD], median, minimum, and maximum.

4.3.3 Primary Efficacy Outcome

The primary efficacy outcome for this study is the percent detection of RLS/PFO with raTCD compared against standard of care Transthoracic Echocardiography (TTE)

4.3.3.1 Primary Efficacy Hypothesis

The primary efficacy hypothesis for the percent detection of RLS/PFO with raTCD against TTE is as follows:

$$H_0: p_{NB-IS\,TCD} = p_{TTE} \;\; vs \; H\_a: p_{NB-IS\,TCD} \neq p_{TTE}$$

1.

4.3.3.2 Primary Efficacy Analysis

We will be utilizing McNemar's Test where 2 x 2 table for raTCD against TTE is:

	Site Assessment Lab TTE Classification	
raTCD Classification	Positive	Negative
Positive	a	b
Negative	c	d

The primary efficacy hypothesis is as follows:

$$H_0: p_b = p_c \;\; vs\; H\_a: p_b \neq p_c$$

4.3.3.3 Sensitivity and Supporting Analyses

Supporting analyses will include the percent agreement rate of RLS/PFO with raTCD vs TTE as well as percent disagreement rate.

The agreement rate will be defined as the percent agreement of true positive and true negative rates for RLS/PFO with raTCD against TTE. We will calculate a Wilson 95% confidence interval.

The difference of positive results will be defined as the percent of false positive and false negative findings for RLS/PFO with raTCD against TTE. We will calculate a Wilson 95% confidence interval.

4.3.4 Secondary Efficacy Outcomes

The secondary efficacy outcomes include:

% detection of false positives, comparison of raTCD with TTE
% detection of true negatives, comparison of raTCD with TTE
% detection of false negatives, comparison of raTCD with TTE
Positive Predictive Value (PPV), comparison of raTCD with TTE
Negative Predictive Value (NPV), comparison of raTCD with TTE
raTCD diagnostic performance as compared to TEE for diagnostic accuracy parameters (sensitivity, specificity, PPV, NPV)
% detection of intervenable shunts, comparison of raTCD with TTE<br>● For raTCD, Grade 3 and above (>30 bubbles) on the Spencer Logarithmic Scale and International Consensus Criteria considered intervenable shunt.<br>● For TTE, >20 bubbles

raTCD No Window rate compared to standard TCD (including both unilateral and bilateral absent transtemporal acoustic windows).
% success rate of raTCD<br>• Incidence of failed registrations (device unable to register)<br>• Incidence of bilateral signals not found (no evaluable signals found both sides)<br>• Incidence of unilateral signals not found (evaluable signal found on one side)
Incidence of device malfunctions

These outcomes will be analyzed in the similar manner as the primary efficacy analysis and supporting analyses.

4.3.5 Exploratory Analysis Endpoint Analyses

Demonstration of raTCD accuracy to Spencer Logarithmic Scale (SLS) Grade and International Consensus Criteria (ICC) grade for diagnostic accuracy.

These outcomes will be analyzed in the similar manner as the primary efficacy analysis and supporting analyses.

5 List of Tables, Figures and Listings

Title	Population
Subject Enrollment and Image Accountability, All Subjects	All Subjects
Subject Demographics, Subjects with raTCD Imaging	ITS Subjects
Medical History, Subjects with raTCD Imaging	ITS Subjects
Stroke Information, Subjects with raTCD Imaging	ITS Subjects
raTCD Imaging Results based on Core Lab Assessment, Subjects with raTCD Imaging	Per-Protocol Subjects
Effect of VM on RLS Diagnosis in Robotic TCD, Subjects with raTCD Imaging	Per-Protocol Subjects
Effect of Bed Position on RLS Diagnosis in Robotic TCD, Subjects with raTCD Imaging	Per-Protocol Subjects
SOC TCD Imaging Results based on Local Assessment, Subjects with raTCD Imaging	Per-Protocol Subjects
SOC TTE and TEE Imaging Results based on Core Lab Assessment and Local Assessment, Subjects with raTCD Imaging	Per-Protocol Subjects
raTCD RLS/PFO Classification Compared to Core Lab Classification based on SOC Imaging, Subjects with raTCD Imaging	Per-Protocol Subjects
raTCD RLS/PFO Classification Compared to Core Lab Classification based on SOC Imaging, All Eligible Subjects	ITS Subjects
raTCD RLS/PFO Classification Compared to SOC TEE Core Lab Assessment, Subjects with raTCD Imaging	Per-Protocol Subjects
raTCD RLS/PFO Classification Compared to SOC TEE Core Lab Assessment, ITT Population	ITS Subjects

raTCD RLS/PFO Classification Compared to SOC TTE Core Lab Assessment, Subjects with raTCD Imaging	Per-Protocol Subjects
raTCD RLS/PFO Classification Compared to SOC TTE Core Lab Assessment, ITT Population	ITS Subjects
raTCD RLS/PFO Classification Compared to Site Assessment based on SOC Imaging, Subjects with raTCD Imaging	Per-Protocol Subjects
raTCD RLS/PFO Classification Compared to Site Assessment based on SOC Imaging, ITT Population	ITS Subjects
raTCD RLS/PFO Classification Compared to SOC TEE Site Assessment, Subjects with raTCD Imaging	Per-Protocol Subjects
raTCD RLS/PFO Classification Compared to SOC TEE Site Assessment, ITT Population	ITS Subjects
raTCD RLS/PFO Classification Compared to SOC TTE Site Assessment, Subjects with raTCD Imaging	Per-Protocol Subjects
raTCD RLS/PFO Classification Compared to SOC TTE Site Assessment, ITT Population	ITS Subjects
raTCD RLS/PFO Classification Compared to SOC TCD Site Assessment, Subjects with raTCD Imaging	Per-Protocol Subjects
raTCD RLS/PFO Classification Compared to SOC TCD Site Assessment, ITT Population	ITS Subjects
raTCD RLS/PFO Classification with SLS Grade Compared to Core Lab Classification based on SOC Imaging, Subjects with raTCD Imaging	Per-Protocol Subjects
raTCD RLS/PFO Classification with SLS Grade Compared to SOC TEE Core Lab Classification based on SOC Imaging, Subjects with raTCD Imaging	Per-Protocol Subjects
High conductance shunts (SLS >=3) raTCD Compared to SOC TTE Core Lab Assessment Bubble Count > 20, Subjects with raTCD Imaging	Per-Protocol Subjects
High conductance shunts (SLS >=3) raTCD Compared to SOC TTE Core Lab Assessment Bubble Count > 20, ITT Population	ITS Subjects
raTCD RLS/PFO Classification with SLS Grade Compared to Site Assessment based on SOC Imaging, Subjects with raTCD Imaging	Per-Protocol Subjects
raTCD RLS/PFO Classification with SLS Grade Compared to SOC TEE Site Assessment based on SOC Imaging, Subjects with raTCD Imaging	Per-Protocol Subjects
High conductance shunts (SLS >=3) raTCD Compared to SOC TTE Site Assessment, Subjects with raTCD Imaging	Per-Protocol Subjects
raTCD RLS/PFO Classification with SLS Grade Compared to SOC TCD Site Assessment based on SOC Imaging, Subjects with raTCD Imaging	Per-Protocol Subjects
