# Supplementary material for: Robot-Assisted Transcranial Doppler Versus Transthoracic Echocardiography for Right to Left Shunt Detection
Source: Stroke. 2023 Oct 5;54(11):2842–50. doi: 10.1161/STROKEAHA.123.043380 (PMC10589435; doi:10.1161/STROKEAHA.123.043380)
Supplement: Supplementary file 4 [file str-54-2842-s004.pdf]

Section & Topic	No	STARD Checklist	Reported on page
**TITLE OR ABSTRACT**			
	**1**	Identification as a study of diagnostic accuracy using at least one measure of accuracy (such as sensitivity, specificity, predictive values, or AUC)	6
**ABSTRACT**			
	**2**	Structured summary of study design, methods, results, and conclusions (for specific guidance, see STARD for Abstracts)	6
**INTRODUCTION**			
	**3**	Scientific and clinical background, including the intended use and clinical role of the index test	5
	**4**	Study objectives and hypotheses	6
**METHODS**			
Study design	**5**	Whether data collection was planned before the index test and reference standard were performed (prospective study) or after (retrospective study)	6
Participants	**6**	Eligibility criteria	6
	**7**	On what basis potentially eligible participants were identified (such as symptoms, results from previous tests, inclusion in registry)	6
	**8**	Where and when potentially eligible participants were identified (setting, location and dates)	6
	**9**	Whether participants formed a consecutive, random or convenience series	6
Test methods	**10a**	Index test, in sufficient detail to allow replication	7
	**10 b**	Reference standard, in sufficient detail to allow replication	7
	**11**	Rationale for choosing the reference standard (if alternatives exist)	7
	**12a**	Definition of and rationale for test positivity cut-offs or result categories of the index test, distinguishing pre-specified from exploratory	7
	**12 b**	Definition of and rationale for test positivity cut-offs or result categories of the reference standard, distinguishing pre-specified from exploratory	7
	**13a**	Whether clinical information and reference standard results were available to the performers/readers of the index test	7
	**13 b**	Whether clinical information and index test results were available to the assessors of the reference standard	7
Analysis	**14**	Methods for estimating or comparing measures of diagnostic accuracy	8
	**15**	How indeterminate index test or reference standard results were handled	7
	**16**	How missing data on the index test and reference standard were handled	7
	**17**	Any analyses of variability in diagnostic accuracy, distinguishing pre-specified from exploratory	8
	**18**	Intended sample size and how it was determined	8
**RESULTS**			
Participants	**19**	Flow of participants, using a diagram	9
	**20**	Baseline demographic and clinical characteristics of participants	9
	**21a**	Distribution of severity of disease in those with the target condition	N/A
	**21 b**	Distribution of alternative diagnoses in those without the target condition	N/A
	**22**	Time interval and any clinical interventions between index test and reference standard	N/A
Test results	**23**	Cross tabulation of the index test results (or their distribution) by the results of the reference standard	9
	**24**	Estimates of diagnostic accuracy and their precision (such as 95% confidence intervals)	9
	**25**	Any adverse events from performing the index test or the reference standard	10
**DISCUSSION**			
	**26**	Study limitations, including sources of potential bias, statistical uncertainty, and generalisability	11

	**27**	Implications for practice, including the intended use and clinical role of the index test	11
**OTHER INFORMATION**			
	**28**	Registration number and name of registry	13
	**29**	Where the full study protocol can be accessed	6
	**30**	Sources of funding and other support; role of funders	6
